# Prospective Whole-Genome Sequencing to Identify Bacterial Transmission and Its Modifiers in Neonates

**DOI:** 10.1001/jamanetworkopen.2025.41409

**Published:** 2025-11-21

**Authors:** Timmy Nguyen, Fabian Bürkin, Stefany Ayala-Montaño, Iván Acevedo Monterrosa, Daniel Jonas, Daniel Klotz, Hans Fuchs, Martin Kuntz, Christian Schneider, Martin Wolkewitz, Tjibbe Donker, Sandra Reuter, Tim Götting, Philipp Henneke

**Affiliations:** 1 Institute for Infection Prevention and Control, Medical Center–University of Freiburg, Freiburg, Germany; 2 Department of General Pediatrics, Adolescent Medicine and Neonatology, Medical Center–University of Freiburg, Freiburg, Germany; 3 Bethel Center for Pediatrics, Department of Neonatology and Pediatric Intensive Care, University Hospital OWL, University of Bielefeld, Bielefeld, Germany; 4 Institute for Medical Microbiology and Hygiene, Medical Center–University of Freiburg, Freiburg, Germany; 5 Institute of Medical Biometry and Statistics, Medical Center–University of Freiburg, Freiburg, Germany

## Abstract

**Importance:**

Infants in neonatal intensive care units (NICUs) are at risk of acquiring organisms with multidrug resistance or high epidemic potential (MDRO+), which may precede invasive infections. High-resolution analysis of transmission cultures of MDRO+ may help mitigate these risks through targeted infection prevention measures.

**Objectives:**

To assess the potential of whole-genome sequencing in resolving suspected transmission chains of MDRO+ and to identify associated risk factors for cluster involvement.

**Design, Setting, and Participants:**

This prospective monocentric cohort study was conducted at a level III NICU at the Medical Center–University of Freiburg, Freiburg, Germany. Of 551 admitted infants, 434 were included because they remained on the ward for 48 hours or more and received 1 or more screenings between February 15, 2019, and November 16, 2020. Statistical analysis was conducted from December 1, 2021, to November 10, 2024.

**Exposures:**

Time-dependent patient- and ward-level factors, medical device use, nursing effort score, invasive procedures, antibiotic use, prevalence of MDRO+, and staffing metrics were analyzed for association with transmission chains.

**Main Outcomes and Measures:**

The primary outcome was transmission of MDRO+, defined as colonization or invasion with genetically indistinguishable bacteria, defined by amplified fragment length polymorphism or whole-genome sequencing. Secondary outcomes included colonization rates, bloodstream infections, and risk factors associated with transmission.

**Results:**

The study included 434 infants (median gestational age, 34.6 weeks [IQR, 31.4-38.3 weeks]; 242 boys [55.8%]; median birth weight, 2165 g [IQR, 1410-2965 g]). Overall, 225 patients (51.8% [95% CI, 47.1%-56.5%]) were colonized with at least 1 MDRO+. Of 418 unique colonizations, 142 (34.0% [95% CI, 29.6%-38.6%]) were linked with transmission by whole-genome sequencing. Thirty-seven unique transmission clusters were identified, most frequently involving *Escherichia coli* (n = 11). Four of 10 bloodstream infections with MDRO+ (40.0%) were linked with transmission events. Increased full-time nurse staffing (odds ratio [OR], 0.28 [95% CI, 0.21-0.38]; *P* < .001) and prior antibiotic use (OR, 0.41 [95% CI, 0.26-0.63]; *P* < .001) were associated with decreased transmission risk, while vascular catheter use was associated with increased transmission risk (OR, 1.65 [95% CI, 1.26-2.17]; *P* < .001).

**Conclusions and Relevance:**

This cohort study involving infants in the NICU suggests that bacterial sequencing can accurately detect bacterial transmission events. Multivariate analysis suggests that the bacterial transmission risk on a NICU can be modified.

## Key Points

**Question:**

Can prospective whole-genome sequencing identify bacterial transmission events and associated factors in a neonatal intensive care unit?

**Findings:**

In this cohort study, 51.8% of participants were colonized with at least 1 bacterial strain with antibiotic resistance or epidemic potential, and 34.0% of colonizations were linked to transmission. Increased full-time nurse staffing and prior antibiotic exposure were associated with a lower risk of transmission-linked colonization, while vascular catheter use was associated with an increased risk.

**Meaning:**

This study suggests that whole-genome sequencing can identify bacterial transmission events and help identify modifying factors for colonization in newborn infants in intensive care.

## Teaser

This cohort study of patients in the neonatal intensive care unit assesses the potential of prospective whole-genome sequencing in resolving suspected transmission chains of organisms with multidrug resistance or high epidemic potential and identifies associated risk factors for cluster involvement.

## Introduction

In healthy infants, the establishment of metabolic independence after birth is associated with acquisition of maternal microorganisms from the birth canal, skin, and breast milk. This acquisition drives the dynamic microbiome development in a postnatal relationship between mother and infant best termed *separate, but intertwined*. However, in hospitalized and, in particular, in preterm infants, the microbiome receives major input from hospital-adapted microorganisms residing in other patients and on inanimate surfaces. Conceptually, these microorganisms are qualitatively distinct, given that they have evolved traits shaped by hosts that carry diseases, by antibiotic selection pressure, and the hospital environment. In contrast with older children and adults, the microbiome of infants is less resilient to the incorporation of new strains of microorganisms. The altered antimicrobial resistance in neonates acutely links microbiome composition and thus hospital-adapted bacteria to life-threatening individual infections and infection outbreaks on neonatal intensive care units (NICUs). A recent study confirmed the difficulty of preventing transmission events in infants even with stringent hygiene measures in a randomized clinical trial of probiotics; more than 48% of infants in the placebo group (ie, without direct contact with the probiotics) eventually carried the probiotic *Bifidobacterium* strain.^1^ Overall, the gradual colonization of infants in NICUs includes bacteria with multidrug resistance (MDR) or increased potential for patient-to-patient transmission.^2,3,4,5^ Nosocomial bacterial colonization, antibiotic use, and indwelling catheters are associated with increased risk of nosocomial infections^6^ and death.^7^ Factors associated with bacterial colonization include prematurity, mode of delivery,^8,9^ antibiotic therapy,^10,11^ and the hospital environment.^12^ In contrast, factors associated with transmission events in NICUs remain largely elusive. The spatial and temporal clustering of bacteria of the same species is suggestive of an outbreak^13^ and should be investigated with appropriate typing methods to confirm or refute putative transmission events.^14^ Despite extensive infection prevention control measures, including environmental investigations, point sources are infrequently discovered,^15,16^ indicating that transmission events predominantly occur via indirect contact (eg, the skin of health care personnel). In Germany, weekly microbiological surveillance has been recommended for infants with very low birth weight (VLBW; <1500 g) by the Robert Koch-Institute since 2012.^17^ This culture-based screening targets potentially problematic bacterial species (ie, species belonging to *Enterobacterales* and nonfermenters declared MDR gram-negative bacteria and methicillin-resistant *Staphylococcus aureus*),^18^ together with their antibiotic-sensitive counterparts, which are collectively referred to as organisms with multidrug resistance or high epidemic potential (MDRO+).^1^ Multidrug-resistant gram-negative bacteria are relatively rare in NICUs in Germany and many other European countries (ie, their parallel, species-identical detection in multiple patients is highly indicative of a transmission event). However, this is not the case for parallel detection of the more frequent non-MDR *Enterobacterales*, because they often represent distinct strains of the same species, yet with similar resistance patterns. Accordingly, the extent of recommended and potentially disruptive organizational infection prevention control measures, ranging from cohorting of patients and staff to environmental sampling and ward closures, is often at odds with inaccurate characterization of MDRO+, in particular, their genomic association. Moreover, a stepwise complex typing of isolates usually introduces considerable delay, so that the necessity of the hygiene measures taken can be assessed only retrospectively. Ultimately, this study aimed to evaluate the frequency of colonization with and transmissions of MDRO+ as a basis for identifying potential factors associated with interindividual bacterial spread.

## Methods

### Design, Setting, and Participants

**Figure 1.**
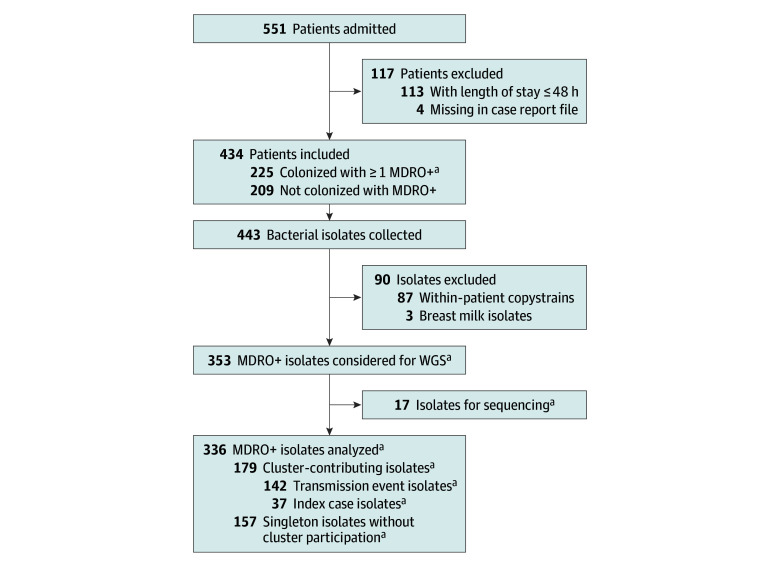
Study Flowchart Bacterial isolates (organisms with multidrug resistance or high epidemic potential [MDRO+]) from weekly screenings of the included patients were used to determine genomic clusters using whole-genome sequencing (WGS). The genomic information was combined with clinical and ward-specific data for multivariate logistic regression analysis. The genomic analysis resolved putative clusters of MDRO+, and multivariate analysis identified factors associated with either increased or decreased risk of becoming part of a transmission cluster within the boundaries of the model. ^a^After copystrain removal (patient, MDRO+, or cluster attribution).

This single-center prospective cohort study was conducted at a 17-bed level III NICU (Medical Center–University of Freiburg, Freiburg, Germany), managing preterm and term newborns. The NICU comprised three 4-bed rooms, one 3-bed room, and one 2-bed room, all without the ability to allow newborns to stay with their parent(s). Parents either stayed at home, were accommodated in a nearby hotel, or were admitted to the obstetrics and gynecology ward. A total of 434 newborns were enrolled from February 15, 2019, to November 16, 2020. Patients admitted to the NICU for 48 hours or more with 1 or more screening were included; those with shorter stays or no screening were excluded. Figure 1 outlines the study design. The initial protocol was published before the study began.^19^ Independent from the study and in accordance with national recommendations,^17^ culture-based screening was performed on admission and on each subsequent Sunday for all patients (eMethods 1 in Supplement 1). This cohort study followed the Strengthening the Reporting of Observational Studies in Epidemiology (STROBE) reporting guideline. The study was approved by the ethics committee of the Medical Center–University of Freiburg on August 28, 2018, with a positive amendment on October 8, 2024. In accordance with the ethical approval, no written or oral consent of study participants was obtained because patient data were deidentified.

### Outcomes

The primary outcome was the identification of bacterial transmission events, defined as indistinguishable same-species pathogens in different patients based on genomic typing. Secondary outcomes included the rate of patients colonized with 1 or more MDRO+, the rate of colonization with MDRO+, and the rate of bloodstream infections (BSIs) per 1000 patient-days. We also investigated factors associated with the risk of being part of a transmission cluster.

### Bacterial Transmission: Definitions According to Whole-Genome Sequencing

A transmission cluster was defined as 2 or more genetically indistinguishable bacterial isolates from different patients, including the index patient. A transmission event was defined as bacterial isolates from 2 or more patients with a difference of fewer than 10 single-nucleotide variations and an epidemiologic link (<60 days’ distance in patient samplings). Singletons were isolates that could not be attributed to transmission clusters and transmission events.

### Collection of Data

We collected patient-level clinical (eTable 1 in Supplement 1) and microbiological data, as well as anonymized nurse staffing data according to the German Federal Joint Committee.^20^ Bacterial screening isolates were transferred from the microbiology department to the typing laboratory. Swab sites included the nasopharynx, anorectal junction, and other risk sites (eg, stomata). For further information on data and bacterial isolate collection, see eMethods 2, 3, and 4 in Supplement 1.

### Genetic Typing of Bacterial Strains

Amplified fragment length polymorphism (AFLP) typing for gram-negative bacteria and *spa*-typing for methicillin-susceptible and methicillin-resistant *S aureus* were performed using Genetic Analyzer abi 3500 and the analytical software GeneMapper (Thermo Fisher Scientific) or BioNumerics (Applied Maths NV). Whole-genome sequencing (WGS) was based on the MiSeq Nextera platform (Illumina). Data are stored in ENA project PRJEB81699^21^ (Supplement 2). A total of 443 isolates were collected. After exclusion of 3 breast milk samples and removal of 87 within-patient duplicate copy strains, 353 nonduplicate isolates of MDRO+ remained. Of these, 179 cluster-contributing isolates entered the transmission analysis, 157 were WGS singletons, and 17 were not sequenced. Among the 179 cluster-contributing isolates, 166 (92.7%) were typed with both AFLP or *spa* typing and WGS and 13 (7.3%) were typed with WGS only. For further information on genetic typing, determination of phylogenetic proximity, quality control parameters, and accessions, see eMethods 5 in Supplement 1.

### Statistical Analysis

Statistical analysis covered the period from December 1, 2021, to November 10, 2024. Analysis was performed with Excel, version 2408 (Microsoft Corp), and R, version 4.4.1 (R Project for Statistical Computing). Outliers were retained. Patient data pseudonymization was performed via hash functions. The proportion of patients colonized with at least 1 MDRO+ was calculated with 95% CIs using the Wilson method. Colonization rates per 1000 patient-days were computed using the Poisson exact test with corresponding 95% CIs. Mismatch rates between AFLP and WGS typing were calculated for cluster-contributing isolates. Within each transmission cluster, the most frequent AFLP type was identified as the majority type, and the mismatch rate was calculated as the proportion of isolates whose WGS cluster definition differed from this majority. Mismatch rates were then averaged by bacterial species to derive a species-specific cumulative mismatch rate. Higher mismatch rates were then associated with lower cluster resolution capabilities. Following Barnett and Graves^22^ and Breslow and Day,^23^ temporal dynamics of the multivariate risk factor analysis for transmission events was mapped using a logistic regression model, which allows a differentiated view of relevant time periods before a transmission event could be detected. Temporal associations between transmission events and evaluated covariates (patient- and ward-specific factors) are shown in eFigure 1 in Supplement 1. The candidate set of covariates used for model construction are listed in the Table and include time-dependent patient-specific and ward-specific factors, such as perinatal characteristics, care practices, invasive procedures, antibiotic use, unit-level prevalence of MDRO+, and staffing metrics. Model fit was assessed using the Akaike information criterion, which guided selection of covariates and their corresponding time windows. This criterion balances model complexity with goodness of fit and is particularly suitable in high-dimensional exploratory analyses (eMethods 6 in Supplement 1). All *P* values were from 2-sided tests and results were deemed statistically significant at *P* < .05.

**Table.** Candidate Set for Potential Covariates by Patient and Day^a^

Variable	Type	Explanation
Individual variables, per patient		
Sex	Binary	Sex of patient
Delivery	Categorical	Type of delivery (emergency cesarean delivery, primary cesarean delivery, secondary cesarean delivery, vaginal delivery)
Birth weight	Continuous	Birth weight in grams
Gestational age	Continuous	Gestational age in weeks
INPULS^b^	Continuous	Based on categorical severity score INPULS in NICUs (treated as a continuous variable to map temporal dynamics via moving averages)
Breast milk	Binary	Yes, 1; and no, 0
Feeding tube	Binary	Yes, 1; and no, 0
Skin-to-skin contact	Binary	Yes, 1; and no, 0
Antibiotics	Continuous	No. of different antibiotics administered, regardless of dose and frequency (ampicillin, tobramycin, piperacillin-tazobactam, meropenem, vancomycin, cefuroxime, erythromycin, rifampicin, cefotaxime, levofloxacin, teicoplanin, linezolid, ceftazidine, trimethoprim-sulfamethoxazole, amphotericin B)
Catheter	Continuous	No. of different catheters (venous [peripheral, central] and arterial)
Invasive ventilation	Binary	Ventilation with breathing tube: yes, 1; and no, 0
Environmental variables, per ward		
MDRO+	Continuous	Total No. of isolated species of MDRO+ per day
Bed occupancy	Continuous	No. of patients, 24-h mean
Nurse to patient ratio	Continuous	24-h Mean
Staff: full-time equivalent	Continuous	Nursing staff totaled over 24 h in full-time equivalents
Staff: deviance	Continuous	Difference between nursing staff number as defined in national guidelines (G-BA) and staff actually present in full-time equivalents, 24-h mean
Staff: understaffed	Continuous	No. of missing full-time nursing staff compared with the requirement determined by G-BA, totaled across all 3 shifts
Staff: overstaffed	Continuous	No. of additional full-time nursing staff exceeding number as defined in national guidelines (G-BA), 24-h mean

Abbreviations: G-BA, German Federal Joint Committee; MDRO+, organisms with multidrug resistance or high epidemic potential; NICU, neonatal intensive care unit.

^a^ Covariates for multivariate analysis in individual (patient-specific) and environmental variables (ward specific). Potential confounders were also considered as equally weighted moving averages over time (up to 14 days in the past).

^b^ INPULS score: Developed by University Hospital Heidelberg, the score is a standardized tool to quantify patients’ nursing care needs by assessing a range of clinical and functional parameters, ranging from category 1 for the lowest care needs to category 6 for the highest.

## Results

### Demographics

This study included 434 study participants of 551 NICU admissions, of which 192 were female (44.2% [95% CI, 39.5%-49.1%]) and 242 were male (55.8% [95% CI, 50.9%–60.5%]), with a median birth weight of 2165 g (IQR, 1410-2965 g) and a median gestational age of 34.6 weeks (IQR, 31.4-38.3 weeks) (eTable 2 in Supplement 1). A total of 113 patients did not meet the inclusion criteria because their length of stay on the ward was less than 48 hours or they had not undergone routine screening. Four patients were excluded due to incomplete data in the case report form. The median length of stay was 29 days (IQR, 13-56 days) for patients with a birth weight less than 1500 g and 6 days (IQR, 3-10 days) for those with a birth weight of 1500 g or more. We observed 7940 patient-days, of which 7033 had complete data for risk factor modeling. Among enrolled patients, 121 (27.9% [95% CI, 23.9%-32.3%]) had a birth weight less than 1500 g (median, 980 g [IQR, 755-1310 g]), a median gestational age of 28.7 weeks (IQR, 26.4-31.0 weeks), and a median length of NICU stay of 29 days (IQR, 13-56 days). Among patients with birth weight less than 1500 g, primary cesarean delivery was the most common delivery mode (63 of 121 [52.1% (95% CI, 42.8%-61.2%)]). A total of 313 patients (72.1% [95% CI, 67.7%-76.1%]) had a birth weight of 1500 g or more, with a median birth weight of 2590 g (IQR, 2050-3280 g), a median gestational age of 36.7 weeks (IQR, 33.86-39 weeks), and a median length of stay of 6 days (IQR, 3-10 days) Among patients with birth weight of 1500 g or more, vaginal birth was the most common delivery mode (124 of 313 [39.6% (95% CI, 34.2%-45.3%)]). Stratification of birth weight showed that lower birth weight was associated with longer hospital stays (eFigure 2 in Supplement 1).

### Colonization

**Figure 2.**
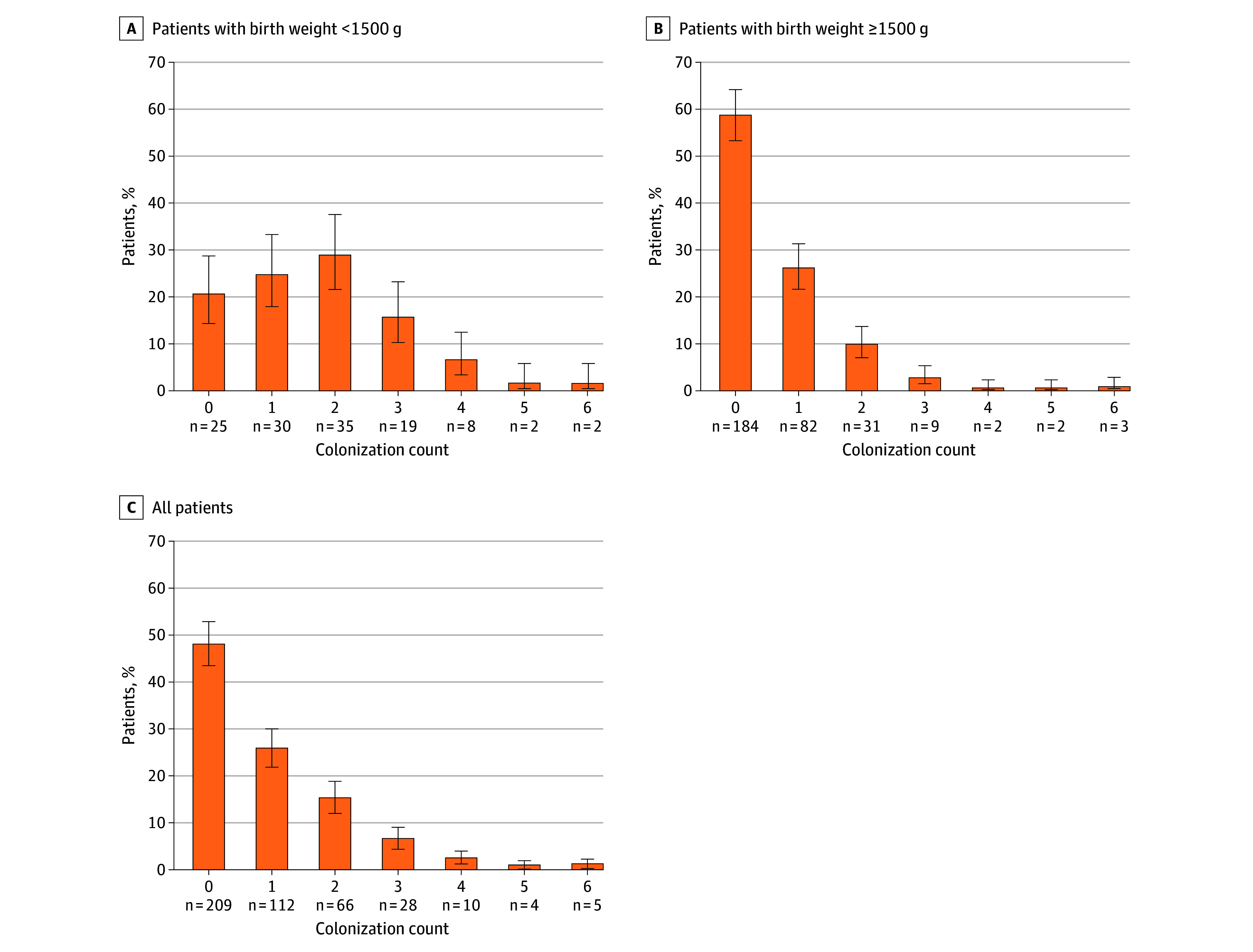
Colonization With Organisms With Multidrug Resistance or High Epidemic Potential (MDRO+) Distribution and counts of patients who experienced no colonizations, single colonizations, or multiple colonizations with MDRO+. Error bars indicate 95% CIs.

Of the 434 enrolled patients, 225 (51.8% [95% CI, 47.1%-56.5%]) were colonized with at least 1 MDRO+ (Figure 2; eTable 2 in Supplement 1). This corresponded to 28.3 patients per 1000 patient-days (95% CI, 24.8-32.3 patients per 1000 patient-days). With 418 colonizations with MDRO+ detected, the overall colonization rate was 52.6 patients per 1000 patient-days (95% CI, 47.7-57.9 patients per 1000 patient-days), regardless of patient assignment. The 5 most frequently detected MDRO+ were *Escherichia coli* (n = 114; 14.4 per 1000 patient-days [95% CI, 12.0-17.2 per 1000 patient-days]), methicillin-sensitive *S aureus* (n = 54; 6.8 per 1000 patient-days [95% CI, 5.2-8.9 per 1000 patient-days]), *Klebsiella oxytoca* (n = 54; 6.8 per 1000 patient-days [95% CI, 5.2-8.9 per 1000 patient-days]), *Enterobacter cloacae* (n = 53; 6.7 per 1000 patient-days [95% CI, 5.1-8.7 per 1000 patient-days]), and *Klebsiella pneumoniae* (n = 48; 6.0 per 1000 patient-days [95% CI, 4.6-8.0 per 1000 patient-days]). All remaining organisms (eg, *Serratia marcescens*, methicillin-resistant *S aureus*, and *Acinetobacter baumannii*) were grouped as “other” and collectively accounted for 12.0 colonizations per 1000 patient-days (n = 95; 95% CI, 9.8-14.6 per 1000 patient-days). Infants with a birth weight less than 1500 g were most commonly colonized by 2 different species of MDRO+; infants with a birth weight of 1500 g or more were most commonly colonized by zero species of MDRO+, with 5 patients colonized with up to 6 different MDRO+. In infants with birth weight of 1500 g or more, MDRO+ were found significantly earlier in screening (median, 4 days [IQR, 2-6 days]) than in infants with birth weight of less than 1500 g (median, 9 days [IQR, 4-15 days]) (eFigure 3A in Supplement 1). Among patients without any colonization with MDRO+, the median length of stay was 4 days (IQR, 2-7 days) for infants with a birth weight of 1500 g or more and 7 days (IQR, 4-10 days) for infants with birth weight less than 1500 g (eFigure 3B in Supplement 1). Species stratification of MDRO+ showed no significant difference in days to first detection per patient (eFigure 4 in Supplement 1).

### Transmission Clusters

A total of 142 of 418 unique colonizations (34.0% [95% CI, 29.6%-38.6%]) were linked to transmission. Whole-genome sequencing identified 37 unique transmission clusters. *E coli* clusters were the most frequent (n = 11), *K oxytoca* formed the largest cluster (20 patients), and *S marcescens* formed a single cluster (7 patients) (eTable 3 in Supplement 1). The transmission event distribution over time is shown in eFigure 5A in Supplement 1. The overall transmission rate of MDRO+ was 17.9 per 1000 patient-days (95% CI, 15.1-21.8 per 1000 patient-days), with *E coli* having the highest single rate of 6.9 per 1000 patient-days (95% CI, 5.2-9.0 per 1000 patient-days) (eFigure 5B in Supplement 1). Whole-genome sequencing identified 157 bacterial isolates not contributing to transmission clusters (ie, singletons) (eTable 4 in Supplement 1); 179 isolates contributed to transmission clusters (eTable 3 in Supplement 1). The phylogeny of *E coli* isolates is shown in eFigure 6 in Supplement 1.

### Discriminatory Capabilities of WGS

We compared precision and validity of AFLP typing and WGS. Of 179 cluster-contributing isolates, 166 (92.7%) were typed with both AFLP or *spa* typing and WGS and 13 (7.3%) were typed with WGS only (after removal of copy strains). For example, the AFLP type D of *E coli* corresponded to sequence type (ST) 141 in all cases, but could be divided into 3 different WGS clusters: 201903_ST141, 201905_ST141, and 201908_ST141 (eFigure 7 and eTable 5 in Supplement 1). Regardless of cluster composition, *E coli* ST 141 was the predominant sequence type (eFigure 8 in Supplement 1), accounting for 28 of 66 cluster-contributing *E coli* isolates (42.4% [95% CI, 31.2%-54.4%]), followed by *K oxytoca* ST 176, accounting for 20 of 38 cluster-contributing *K oxytoca* isolates (52.6% [95% CI, 37.3%-67.5%]).

**Figure 3.**
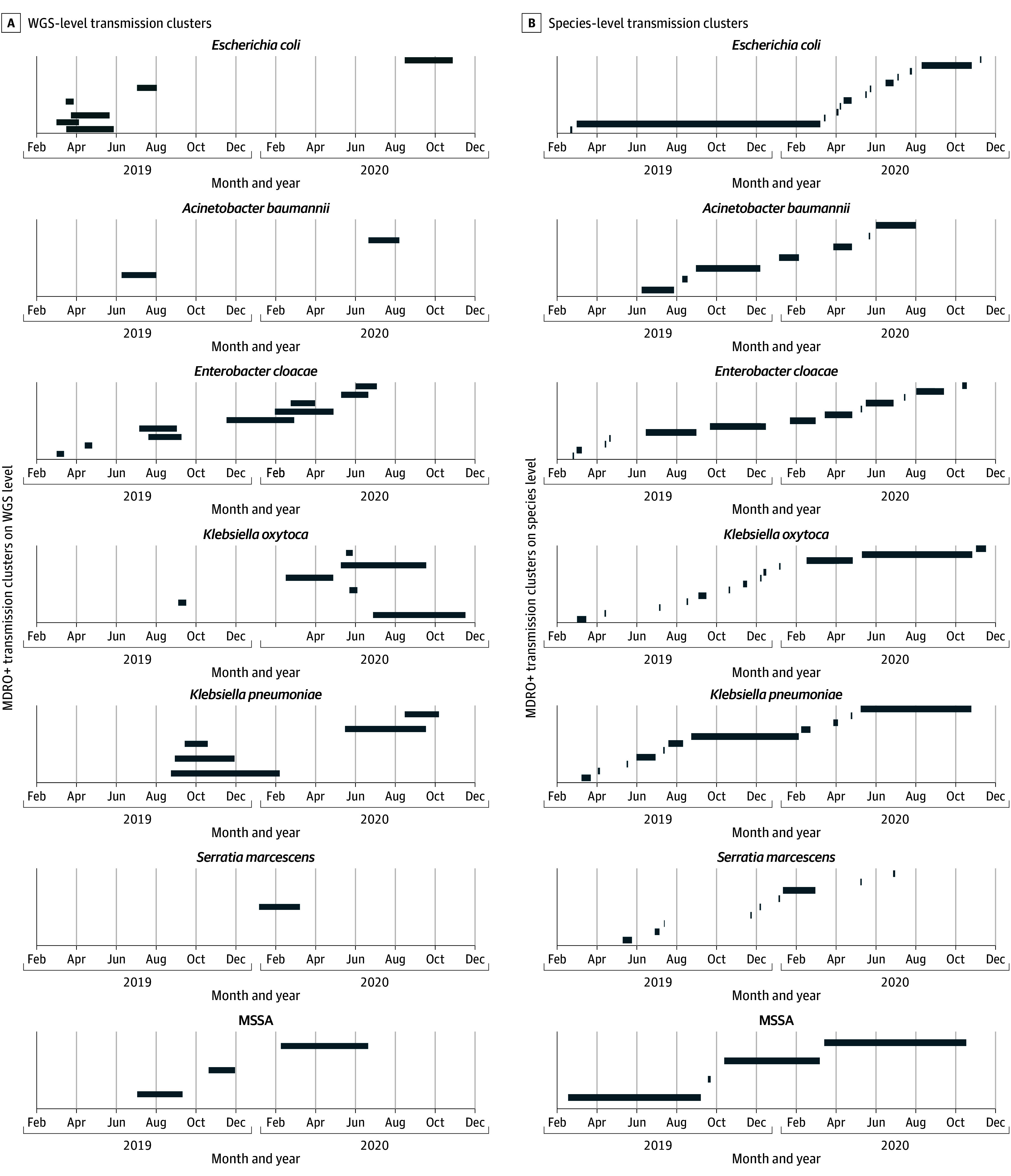
Variable Cluster Definitions A, Cluster definition according to whole-genome sequencing (WGS); the combined timeframes of patients colonized with cluster-contributing isolates were depicted over time and are indicated by horizontal bars. The day of the index case detection defines the start of the timeline. For respective clusters, the discharge of the last patient colonized with a cluster-contributing isolate defined the end of the timeframe. Singletons (non–cluster-contributing isolates) were excluded from this representation. B, Cluster definition according to species alone; overlapping timeframes of both figures for the respective multidrug-resistant organism with epidemic potential indicated a higher resolution in favor of WGS compared with cluster determination based on species alone, which is best illustrated by *Escherichia coli*. MDRO+ indicates organisms with multidrug resistance or high epidemic potential; and MSSA, methicillin-susceptible *Staphylococcus aureus*.

When comparing AFLP typing with WGS cluster identification, mismatch rates between the methods were 0.10 for *K oxytoca*, 0.09 for *E cloacae*, and 0.06 for *E coli* (eTable 6 in Supplement 1). Same-species MDRO+ can cluster differently depending on the strain definition. For example, *E coli* cluster 2 (longest bar for *E coli* in Figure 3B) was defined as the temporal overlap of patients colonized with *E coli* on the ward and formed a large cluster that can be further divided into distinct clusters as defined by WGS (Figure 3A; eTable 7 in Supplement 1), leading to a resolution gain of 9 clusters. Increases in cluster resolution were observed for other species as well (eg, *K oxytoca* cluster 12; eTable 7 in Supplement 1).

### BSIs With MDRO+

Ten BSIs with *E coli*,^5^ *K pneumoniae*,^3^ and *K oxytoca*^2^ were detected. Four patients had BSIs derived from a transmission event (as revealed by WGS), of which 3 were detected with a same-strain MDRO+ before and admitted with a birth weight of less than 1000 g (eFigure 9 in Supplement 1). The BSI isolates belonged to WGS clusters 201907_ST73, 201911_ST73, 201908_ST219, and 202006_ST966 (eTable 3 in Supplement 1). The overall rate of BSIs was 1.3 per 1000 patient-days (95% CI, 0.6-2.3 per 1000 patient-days) and the rate of BSIs resulting from a transmission event was 0.5 per 1000 patient-days (95% CI, 0.1-1.3 per 1000 patient-days).

### Antibiotic Use

Infants with VLBW received selected antibiotics or their combinations proportionally and significantly more often than infants without VLBW (eFigure 10A in Supplement 1). However, the 2 subgroups did not differ in the total number of antibiotic administrations (eFigure 10B in Supplement 1).

### Multivariate Model Analysis

**Figure 4.**
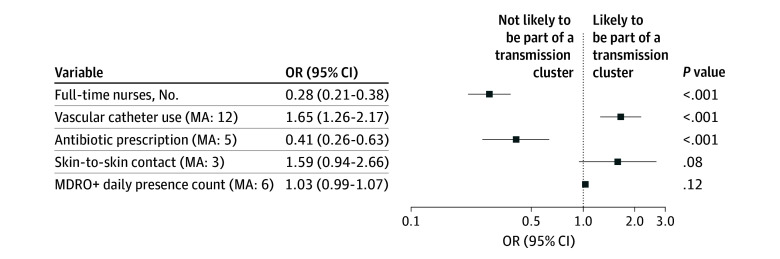
Multivariate Logistic Regression Model Forest plot of adjusted odds ratios (ORs) and 95% CIs derived from a multivariate logistic regression model estimating the likelihood of being part of a transmission cluster. Odds ratios are plotted on a logarithmic scale. Variables incorporating temporal associations are represented using moving averages (MAs) over 3 to 12 days, as indicated. The model was a generalized linear model (binomial family, logit link) with a significance of χ^2^_5_ = 118.06; *P* < .001, Cragg-Uhler pseudo-*R*^2^ = 0.10; McFadden pseudo-*R*^2^ = 0.10. The included number of observations was 7033, with a log likelihood of −556.54. The Akaike information criterion was 1125.08, the corrected Akaike information criterion was 1125.09, and the bayesian information criterion was 1166.23. MDRO+ indicates organisms with multidrug resistance or high epidemic potential.

Multivariate analysis combined patient, genomic, clinical, and staffing data. The logistic regression model estimated the probability of becoming part of a transmission event (outcome variable) based on 5 variables (Figure 4). Of these, 3 variables were significantly associated with the outcome variable. An increase in the number of full-time nurses by 1 unit decreased the odds of a transmission event by 72% (odds ratio [OR], 0.28 [95% CI, 0.21-0.38]; *P* < .001). An increase in antibiotic prescriptions (moving average, 5) decreased the odds of a transmission event by 59% (OR, 0.41 [95% CI, 0.26-0.63]; *P* < .001). An increase in vascular catheter use (moving average, 12) increased the odds of a transmission event by 65% (OR, 1.65 [95% CI, 1.26-2.17]; *P* < .001).

Skin-to-skin contact (moving average, 3) and daily presence count of MDRO+ (ie, colonization pressure; moving average, 6) were not significantly associated with transmission events (Figure 4). With *P* < .001 for χ^2^_5_, the final model outperformed the null model. However, overall fit was weak to moderate, as indicated by limited pseudo-*R*^2^ (McFadden and Cragg-Uhlmann). Variance inflation factor tests indicated no multicollinearity. A rainbow test for linearity indicated that not all logistic regression assumptions were met, limiting the results’ significance.

## Discussion

This study identifies prospective WGS of routine screening isolates from newborns in intensive care as a tool to detect and track transmission chains with MDRO+ at the highest precision. The transmission of—in part, hospital-adapted—microorganisms between patients is commonly regarded as a failure of basic hygiene procedures, especially in individuals at high risk, such as extremely premature infants. However, because very preterm infants typically depend on life-supporting measures immediately after birth, separation from the mother is the norm (ie, microbiome development relies largely on input from staff skin and inanimate surfaces).^24^ Based on our high-resolution genomic surveillance we hypothesize that bacterial transmission frequently occurs on NICUs. Bacterial transmission of hospital-adapted bacteria between patients is generally considered an adverse outcome from an infection prevention control perspective. Failure to comply with infection prevention control measures may promote the emergence of bacterial strains that harbor traits facilitating interindividual spread, in-host expansion, and tissue invasion. This may lead to NICU outbreaks that are particularly feared, especially when bacteria carry resistance against first-line antibiotics.^25^ It seems reasonable that real-time knowledge of the number of patients carrying a particular bacterial strain, the length of time the strain has been in a NICU, and the number of infants who develop clinical symptoms associated with strain carriage will enable the risk of transmission clusters to be calculated. Accordingly, accuracy and speed in bacterial typing should substantially improve accuracy of outbreak prediction.^16^ However, due to routine turnaround times for bacterial typing (including material shipment, DNA preparation, and typing), infection prevention control measures are usually initiated immediately after the appearance of clusters of the same species (ie, before typing results are available) and typing is often not initiated. Our surveillance findings align with national species-level data, emphasizing gram-negative bacteria (especially *E coli*, *E cloacae*, *K oxytoca*, and *K pneumoniae*) as key screening targets and causes for nosocomial infections.^5,26^ Conceptually, cluster definition only on the species level will frequently mischaracterize transmission events. The resulting erroneous declaration of outbreaks may initiate interventions that have the potential to compromise the overall quality of care. Examples are the rejection of new admissions or even the closure of wards.^27,28^ We found AFLP typing to detect transmission events in most cases, similar to what has been reported for Fourier-transformed, infrared spectroscopy–based typing.^29^ However, both methods have limitations in cluster characterization and longitudinal surveillance, including considerable mismatch rates. Accordingly, we asked whether genomic cluster definition by WGS, the criterion standard for bacterial strain discrimination,^30,31^ is associated with a clinical advantage in a level III NICU. We found that WGS revealed AFLP-defined transmission clusters as pseudoclusters. For example, *E coli* formed multiple transmission clusters of ST 73 and ST 141, which, on in-depth phylogenetic analysis with WGS, fell into distinct clusters, highlighting the advantage associated with WGS compared with methods that focus on housekeeping genes. In selected cases, WGS delineated the “microbial biography” from admission, to same-strain colonization and occurrence in clinical samples (blood and tracheal aspirates). The precision in transmission cluster definition by WGS enabled us to identify factors in multivariate analysis associated with transmission. The addition of only 1 full-time nurse on the ward was associated with a decreased risk of patients becoming part of a transmission cluster. This finding seems by no means self-evident, because increasing personnel on a NICU may increase transmissions as well (eg, due to more procedures, longer incubator opening times). Staffing data included nurses declared as state examined with high qualification and did not account for nurses in training. Health care professionals with other training levels and professions should be included in further research. The presence of an indwelling catheters (arterial, venous) in patients was associated with an increased risk of becoming part of a transmission cluster. We hypothesize that vascular catheter presence may be linked with increased patient contact with staff (eg, catheter care). Finally, prior administration of antibiotics was associated with a reduced risk of being part of a transmission cluster. Several factors may underlie this interrelation. The culture-based screening method, preceding typing, may be impacted by antibiotics decreasing the bacterial density at the swabbed areas, as shown for intestinal colonization.^32,33^ These considerations are in line with the fact that infants with VLBW received more antibiotics and first colonization with MDRO+ was detected later compared with patients without VLBW. Given the evidence highlighting the potential short-term and long-term effects of antibiotics (eg, an increased risk for bronchopulmonary dysplasia,^34^ necrotizing enterocolitis or invasive fungal infections,^35^ atopy, asthma, and even mortality), this finding needs exploration in independent studies. In contrast with the outlined factors, the number of patients colonized with MDRO+ on the NICU (colonization pressure), skin-to-skin contact, and the INPULS score (a standardized tool to quantify patients’ nursing care needs by assessing a range of clinical and functional parameters, ranging from category 1 for the lowest care needs to category 6 for the highest care needs)^36^ were not significantly associated with transmission clusters.

### Limitations

This study has some limitations. The swab-based screening in NICUs has limited sensitivity for the detection of colonizing bacteria (eg, because the recommended minimal handling of the most fragile very preterm infants may impact the sampling procedure).^37^ This study did not measure the association of antibiotic administration with bacterial screening sensitivity. Bacterial isolates were cultured on solid media and colonies picked based on morphologic characteristics, potentially missing mixed strains of the same species. With respect to factors that modify transmission events, the data of our hand hygiene compliance observations were not granular enough for multivariate analysis. Although our final model assumed a linear association between risk factors and outcome, the necessary conditions for this linearity could not be met. However, we did not consider nonlinear alternatives such as fractional polynomials,^38,39,40^ splines,^41^ or nonlinear distributed lag models,^42^ as they are considerably more complex or less robust and thus harder to interpret. Finally, the advantages of single-center study (eg, highly standardized procedures and thus comparability between groups) comes with the cost of limited transferability to other NICUs, in particular on the international level.

## Conclusions

In this cohort study, we found integration of WGS with colonization screening to be of substantial value in systematically resolving transmission chains of MDRO+ with the highest precision. Prospective inclusion of granular patient and staffing data with multivariate model analysis builds a foundation for rational, data-driven, and actionable conceptualizations of infection prevention control measures in the future.

## Notes

Supplement 1. **eMethods 1.** Routine Cultural Diagnostics **eMethods 2.** Collection of Patient Data **eMethods 3.** Collection and Preparation of Isolates **eMethods 4.** Collection of Nurse Staffing Data **eMethods 5.** Genetic Typing of Bacterial Strains **eMethods 6.** Statistical Analysis **eFigure 1.** Temporal Relationships Between the Detection of Transmission Events and Potential Influencing Variables Xi **eFigure 2.** Length of Stay of Patient Population **eFigure 3.** MDRO+ Colonization Dynamics on Patient Level **eFigure 4.** MDRO+ Colonization Dynamics on Species Level **eFigure 5.** MDRO+ Transmissions **eFigure 6.** Phylogeny of *E.coli* Isolates **eFigure 7.** Cluster Attribution Comparison (AFLP vs WGS) **eFigure 8.** Cluster Stratification by Sequence Type **eFigure 9.** Alluvial Diagram of Observed MDRO+ Blood Stream Infections **eFigure 10.** Antibiotic Administration by Birth Weight Groups **eTable 1.** Case-Report-Form **eTable 2.** Demographic Data of Study Population **eTable 3.** WGS-Cluster-Contributing MDRO+ **eTable 4.** WGS-non-Cluster-Contributing MDRO+ ("singletons") **eTable 5.** AFLP-Mismatches for *E. coli* **eTable 6.** Cumulative AFLP-Mismatch Rates per MDRO+ **eTable 7.** WGS Enabled Net Gain in Resolution **eAppendix.** Abbreviations
eReferences.

Supplement 2.
Whole-Genome Sequencing Data

Supplement 3.
Data Sharing Statement
