# Supplementary material for: Prospective Whole-Genome Sequencing to Identify Bacterial Transmission and Its Modifiers in Neonates
Source: JAMA Netw Open. 2025 Nov 21;8(11):e2541409. doi: 10.1001/jamanetworkopen.2025.41409 (PMC12639485; doi:10.1001/jamanetworkopen.2025.41409)
Supplement: Supplement 1. — eMethods 1. Routine Cultural Diagnostics eMethods 2. Collection of Patient Data eMethods 3. Collection and Preparation of Isolates eMethods 4. Collection of Nurse Staffing Data eMethods 5. Genetic Typing of Bacterial Strains eMethods 6. Statistical Analysis eFigure 1. Temporal Relationships Between the Detection of Transmission Events and Potential Influencing Variables Xi eFigure 2. Length of Stay of Patient Population eFigure 3. MDRO+ Colonization Dynamics on Patient Level eFigure 4. MDRO+ Colonization Dynamics on Species Level eFigure 5. MDRO+ Transmissions eFigure 6. Phylogeny of E.coli Isolates eFigure 7. Cluster Attribution Comparison (AFLP vs WGS) eFigure 8. Cluster Stratification by Sequence Type eFigure 9. Alluvial Diagram of Observed MDRO+ Blood Stream Infections eFigure 10. Antibiotic Administration by Birth Weight Groups eTable 1. Case-Report-Form eTable 2. Demographic Data of Study Population eTable 3. WGS-Cluster-Contributing MDRO+ eTable 4. WGS-non-Cluster-Contributing MDRO+ ("singletons") eTable 5. AFLP-Mismatches for E. coli eTable 6. Cumulative AFLP-Mismatch Rates per MDRO+ eTable 7. WGS Enabled Net Gain in Resolution eAppendix. Abbreviations eReferences. [file jamanetwopen-e2541409-s001.pdf]

Supplementary Online Content

Nguyen T, Bürkin F, Ayala-Montaño S, et al. Prospective whole-genome sequencing to identify bacterial transmission and its modifiers in neonates. *JAMA Netw Open.* 2025;8(11):e2541409. doi:10.1001/jamanetworkopen.2025.41409

eMethods 1. Routine Cultural Diagnostics
eMethods 2. Collection of Patient Data
eMethods 3. Collection and Preparation of Isolates
eMethods 4. Collection of Nurse Staffing Data
eMethods 5. Genetic Typing of Bacterial Strains
eMethods 6. Statistical Analysis
eFigure 1. Temporal Relationships Between the Detection of Transmission Events and Potential Influencing Variables Xi
eFigure 2. Length of Stay of Patient Population
eFigure 3. MDRO+ Colonization Dynamics on Patient Level
eFigure 4. MDRO+ Colonization Dynamics on Species Level
eFigure 5. MDRO+ Transmissions
eFigure 6. Phylogeny of *E. coli* Isolates
eFigure 7. Cluster Attribution Comparison (AFLP vs WGS)
eFigure 8. Cluster Stratification by Sequence Type
eFigure 9. Alluvial Diagram of Observed MDRO+ Blood Stream Infections
eFigure 10. Antibiotic Administration by Birth Weight Groups
eTable 1. Case-Report-Form
eTable 2. Demographic Data of Study Population
eTable 3. WGS-Cluster-Contributing MDRO+
eTable 4. WGS-non-Cluster-Contributing MDRO+ ("singletons")
eTable 5. AFLP-Mismatches for *E. coli*
eTable 6. Cumulative AFLP-Mismatch Rates per MDRO+
eTable 7. WGS Enabled Net Gain in Resolution
eAppendix. Abbreviations
eReferences.

This supplementary material has been provided by the authors to give readers additional information about their work.

© 2025 Nguyen T et al. *JAMA Network Open.*

eMethods 1. Routine Cultural Diagnostics

Screening swabs (eSwab, Copan, Brescia, Italy) and clinical samples were inoculated on both selective and non-selective media including the following agar plates: Columbia Blood, MacConkey, Hematin Chocolate Agar, chromID® (Biomérieux, Nuertingen, Germany) Extended spectrum beta lactamase (ESBL) and Methicillin-resistant *S. aureus* selective. Media were incubated under aerobic conditions for 48 hours at 36°C and with 5% CO_2. If growth on plates was detected, matrix-assisted-laser-desorption-ionization-time-of-flight (MALDI-TOF) mass spectrometry (Bruker Daltonics, Bremen, Germany) was employed to identify bacterial species. Susceptibility testing was performed using VITEK2® (Biomérieux, Nuertingen, Germany) and/or minimum inhibitory concentration (MIC) test strips (Liofilchem, Piane Romano, Italy) respectively. The results were interpreted according to European Committee on Antimicrobial Susceptibility Testing (EUCAST) clinical breakpoints. Presence of resistance genes was confirmed by nucleic acid amplification tests for carbapenem-resistant gram-negative bacteria and Methicillin-resistant *S. aureus*-.

© 2025 Nguyen T et al. *JAMA Network Open.*

eMethods 2. Collection of Patient Data

We collected clinical data from patient charts or electronic systems (laboratory information system, clinical information system) including patient- and ward-specific data, potential risk factors and confounders as well as procedural and operational data. Information on the case-report-form is provided (*eTable 1*). Outliers and influential values were not corrected or excluded, as extreme situations were considered particularly interesting for the analysis due to everyday hospital hygiene practice and their clinical relevance.

© 2025 Nguyen T et al. *JAMA Network Open.*

eMethods 3. Collection and Preparation of Isolates

If multidrug resistant organism plus (MDRO+) species were found, bacterial isolates were transferred to the typing laboratory. The study included the following bacteria in our genetic and statistical analysis and are referred to as "MDRO+", specifically: *A. baumannii, C. freundii, E. coli, E. cloacae, K. oxytoca, K. pneumonia, P. mirabilis, S. marcescens, P. aeruginosa* and *S. aureus* (methicillin sensitive *Staphylococcus aureus* [MSSA], MRSA) on the basis of the initially published study protocol [1].

eMethods 4. Collection of Nurse Staffing Data

Procedural data contained anonymized daily and shift-specific metrics on bed occupancies, nurse staffing levels and application of minimum staffing criteria. The patients' birth weight, maturity level and complexity of intensive care treatment or intensive care monitoring guided definitions of 'overstaffing' and 'understaffing' at shift level, which are based on algorithms predefined by the Federal Joint Committee, a national regulatory authority which issues mandatory directives on above mentioned criteria (G-BA, German: "Gemeinsamer Bundesausschuss") [2].

© 2025 Nguyen T et al. *JAMA Network Open.*

eMethods 5. Genetic Typing of Bacterial Strains

Amplified fragment length polymorphism (AFLP) typing for gram-negative bacteria, *spa*-typing for *S. aureus* (MSSA, MRSA) and Whole-genome sequencing (WGS) were performed for typing of MDRO+.

AFLP

AFLP typing from pure bacterial cultures was performed with the Genetic Analyzer abi3500. The comparison of the resulting fragment patterns was software-assisted (abi3500 analysis software *GeneMapper*™ [Thermo Fisher Scientific], *BioNumerics* [Applied Maths NV]). The assignation of fragment differences to genotypes was performed as described [3,4].

WGS

Deoxyribonucleic acid (DNA) extraction was performed with a High Pure polymerase chain reaction (PCR) Template (Roche) following standard protocol. Sequencing was carried out using the Illumina MiSeq Nextera DNA Flex Library pre-preparation and V2 300 cycle PE kit according to manufacturer's instructions. Mapping of raw reads was carried out using smalt [5], samtools v0.1.19 [6] and GATK [7] to species-specific reference genomes (*Acinetobacter baumann*ii ST1: A1 (GenBank accession number CP010781), *Escherichia coli* ST131: EC958 (HG941718), *Klebsiella oxytoca* ST19: NCTC11696 (ERS530425), *Klebsiella pneumoniae* ST38 : MGH78578 (CP000647), *Enterobacter cloacae* ST1 : ATCC13047 (CP001918), *Staphylococcus aureus* ST22: HO50960412 (HE681097)). Assembly was carried out with SPAdes v3.13.1 [8] with kmer sizes 21, 33, 55, 77, 99, 109, and 123, with subsequent filtering to only include contigs with a minimum of 500bp. Species identification was carried out on raw reads using kraken v1.1.1 [9]. Multilocus sequence typing (MLST) was carried out on the assemblies using mlst v2.10 [10]. Quality control parameters were a minimum of 30x coverage, appropriate length of the sequence, number of contigs <500, N50 <100,000bp, correct species and MLST identification. Phylogenetic reconstruction was based on core genome alignment to the assembly of a within-cluster reference, identification of single nucleotide polymorphism (SNP) with snp-sites v2.5.1, followed by estimation using RAxML v8.2.12 (default parameters, 100 rapid bootstrapping, GTR+GAMMA) and pairwise distance calculation by snp-dists v0.6. iTOL was used for phylogenetic visualization. Sequencing data has been deposited in the ENA project PRJEB81699 and individual accession identifiers can be found in the supplemental material (Accessions_PRJEB81699.xlsx.

If bacterial isolates were <10 SNPs different (see eTable 3) [11,12] and were collected with temporal proximity (defined as an epidemiological link of less than 60 days between positive sampling dates) we defined them as resulting from transmission events. The first chronological appearance of an isolate in a cluster was regarded as the index case. The minimum number of indistinguishable bacterial isolates required to form a cluster was two.

© 2025 Nguyen T et al. *JAMA Network Open.*

eMethods 6. Statistical Analysis

Following Barnett & Graves [13] and Breslow et al. [14], temporal dynamics of the multivariate risk factor analysis for transmission events (TE) was mapped using a logistic regression model, which allows a differentiated view of relevant time periods before a TE could be detected. We also analyzed mixed models. However, the analysis was conducted based on logistic models since random parts (patients, time and both) were not significant.

Dependent Variable Y_t: Transmission Events

The dependent variable Y_{t+1} is a binary representation of the TE at time $t+1$ ($TE_{(t+1)}$) at the individual patient level with a value of 1 for a day with positive MDRO+ screening and 0 on days with negative or no MDRO+ screening. The postponement of Y in the form of $t+1$ instead of t has practical reasons: If a screening is positive on day $t+1$, with the incubation period the transmission took place on day t at the latest, especially since a large proportion of the screenings took place in the morning.

Independent Variables $X_{(i, t)}$

The candidate set for potential confounders $X_{(i, t)}$ with i *in* $\{1, ..., 18\}$ in the model included 18 variables (*eTable 2*). The variables $X_{(i, t)}$ were also considered as equally weighted moving averages (MA) over the time periods *[t; t], [t; t-1], [t; t-2], ..., [t; t-14]*, so that a total of 15 temporally nuanced variants of each variable were available for selection.

For d in $\{0,1, ..., 14\}$, the respective moving average was defined as:

$$\text{MA: d} := \sum_{j=t}^{t-d} \frac{X_{(i,j)}}{d+1}.$$

In our model e.g. "MA: 2" represents the unweighted average over the two days t, the previous day $t-1$ and $t-2$ (*Figure 2*).

An optimal model was chosen using minimum Akaike's information criterion (AIC). Predictors were selected via stepwise forward-backward selection. Starting from a null model, all univariate models were tested, and the best combination of a confounder and moving average (yielding lowest AIC) was selected. To ensure interpretability, we excluded all alternative moving averages of that predictor among all available variables. If several variants of predictors measured the same target variable (e.g. staff occupancies), only one variable was retained. After adding a new predictor, each included variable was re-evaluated using a 'leave-one-out' approach to seek a lower AIC score alternatives. This process was repeated until no further improvement was possible after including at least two additional variables. The final model, with the lowest AIC, was then tested for multicollinearity (using variance inflation factor [VIF]) and linearity (using a rainbow test [15,16]) to meet logistic regression conditions.

Conditions for logistic regression

One of several necessary conditions for logistic regression is that of linearity. For our model, this condition is checked by performing the rainbow test. In order to restore the condition of linearity when the rainbow test is significant, transformations such as Box-Cox [17] or Yeo-Johnson [18] would be necessary from a technical perspective. However, back transformations of the variables are not valid and the cumbersome interpretations of the transformations prevent a meaningful application in clinical practice. For this reason, even if the rainbow test is significant, no further measures are taken in favor of the interpretability of the results, even if this limits the validity of the model itself.

© 2025 Nguyen T et al. *JAMA Network Open.*

eFigure 1. Temporal Relationships Between the Detection of Transmission Events and Potential Influencing Variables Xi

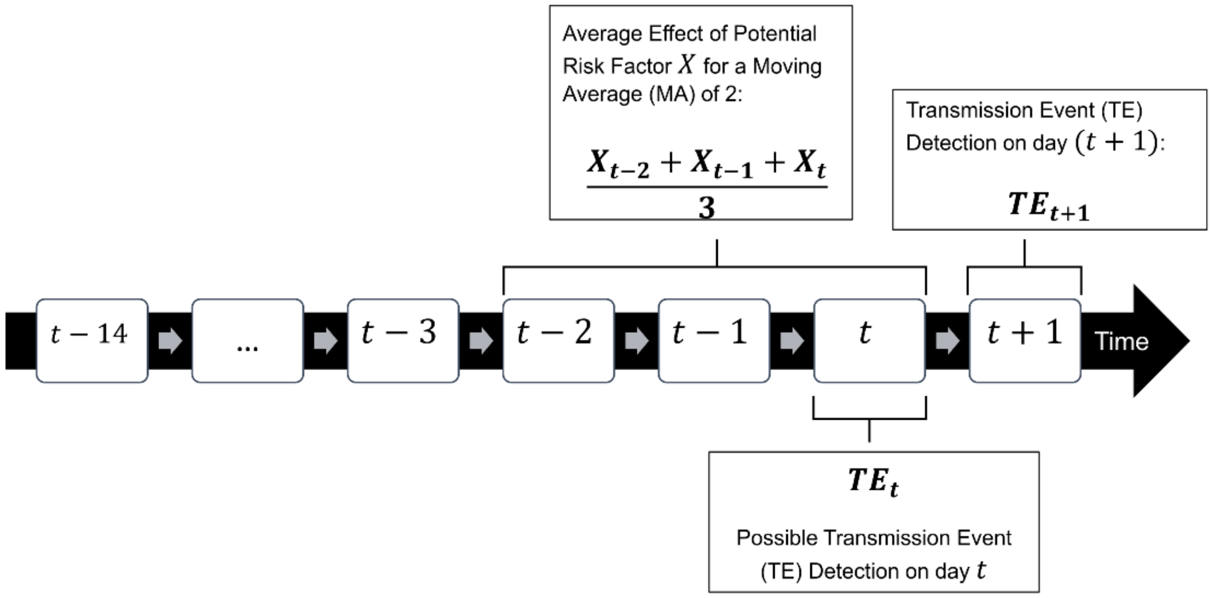

The variable TEs (transmission events) can only determine past transmissions, but not the exact time of the TE. In order to consider this measurement's inaccuracy, the various independent variables X_i are also considered in different time periods with the aid of moving averages (MA).

© 2025 Nguyen T et al. *JAMA Network Open.*

eFigure 2. Length of Stay of Patient Population

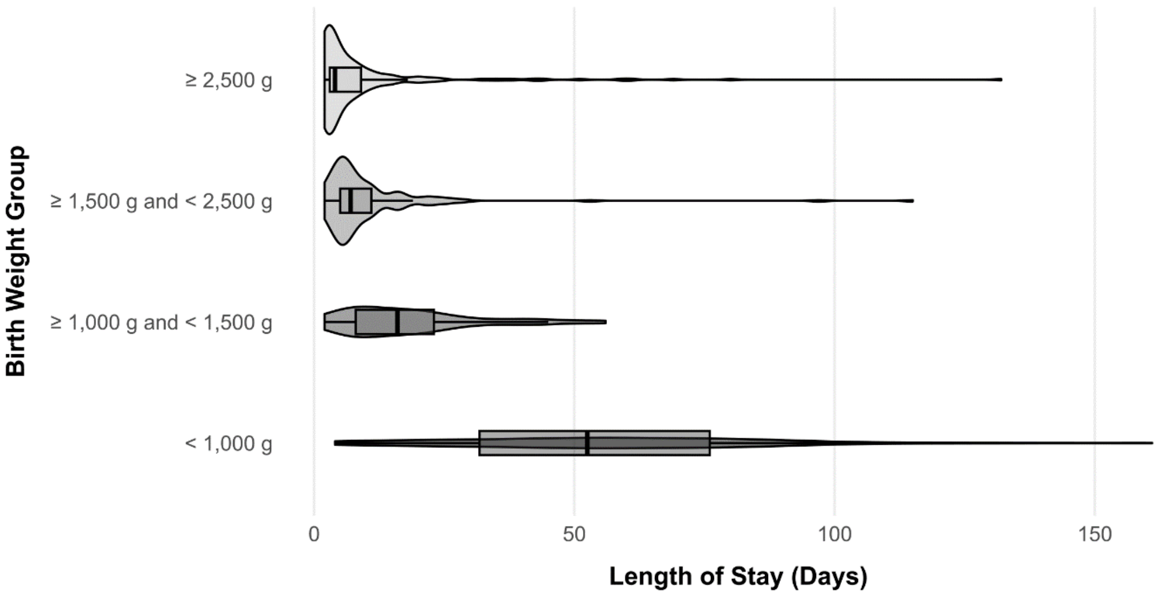

Patient length of stay in days, stratified by birth weight categories.

© 2025 Nguyen T et al. *JAMA Network Open.*

eFigure 3. MDRO+ Colonization Dynamics on Patient Level

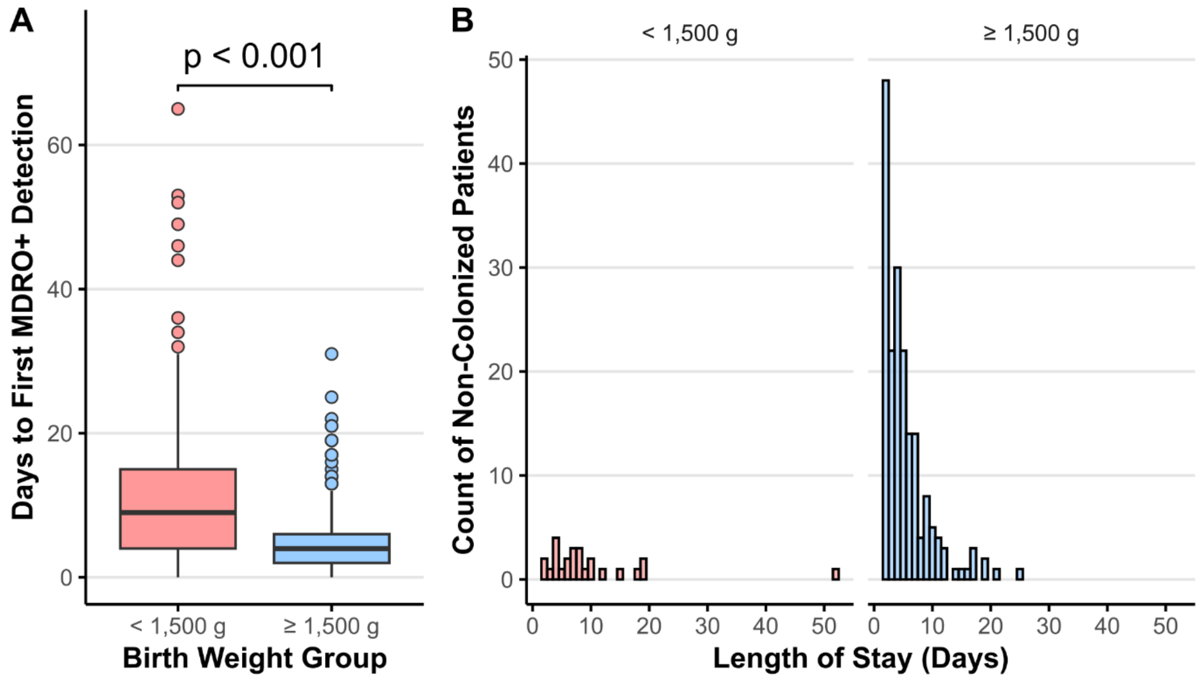

A) Number of days from admission to first MDRO+ detection (regardless of species) by birth weight groups. Patients with birth weight ≥ 1,500 g were colonized with their first MDRO+ significantly earlier (Wilcoxon Rank-Sum Test, $p < 0.001$). **B)** Distribution of length of stay of patients that were not colonized by MDRO+ and stratification by birth weight.

© 2025 Nguyen T et al. *JAMA Network Open.*

eFigure 4. MDRO+ Colonization Dynamics on Species Level

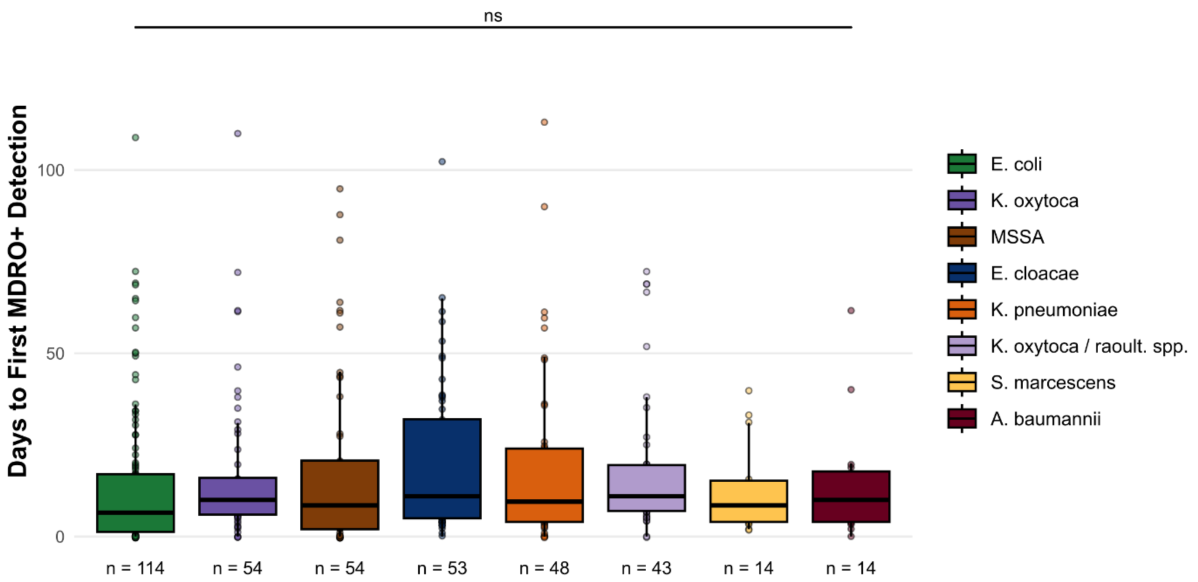

Number of days from admission to first MDRO+ detection (stratified by MDRO+ species). The comparison of MDRO+ showed no statistically significant differences across all combinations of MDRO+ for the time to first detection (Kruskal-Wallis chi-squared = 11.341, df = 7, p = 0.124).

© 2025 Nguyen T et al. *JAMA Network Open.*

eFigure 5. MDRO+ Transmissions

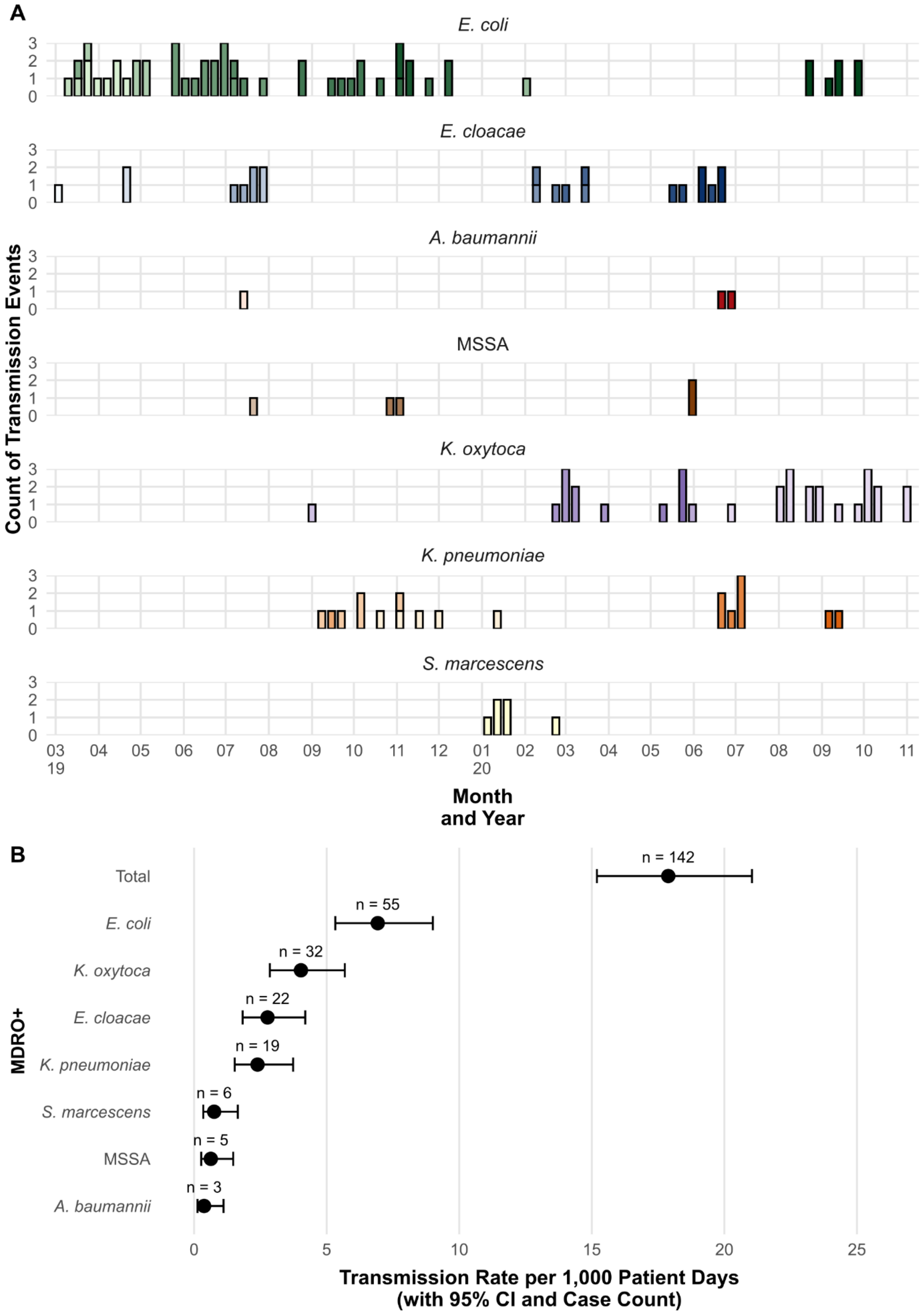

© 2025 Nguyen T et al. *JAMA Network Open.*

A) Occurrence of transmission events over time, stratified by MDRO+ species. Every MDRO+ species is displayed in their respective base colors. Shades of colors indicate the attribution to separate cluster as defined by WGS. Cluster-index-cases were excluded in this representation. **B)** Transmission rate per 1,000 patient days with 95% CIs and category attribution. Stratification by MDRO+ species and in total with respective absolute counts.

© 2025 Nguyen T et al. *JAMA Network Open.*

eFigure 6. Phylogeny of *E.coli* Isolates

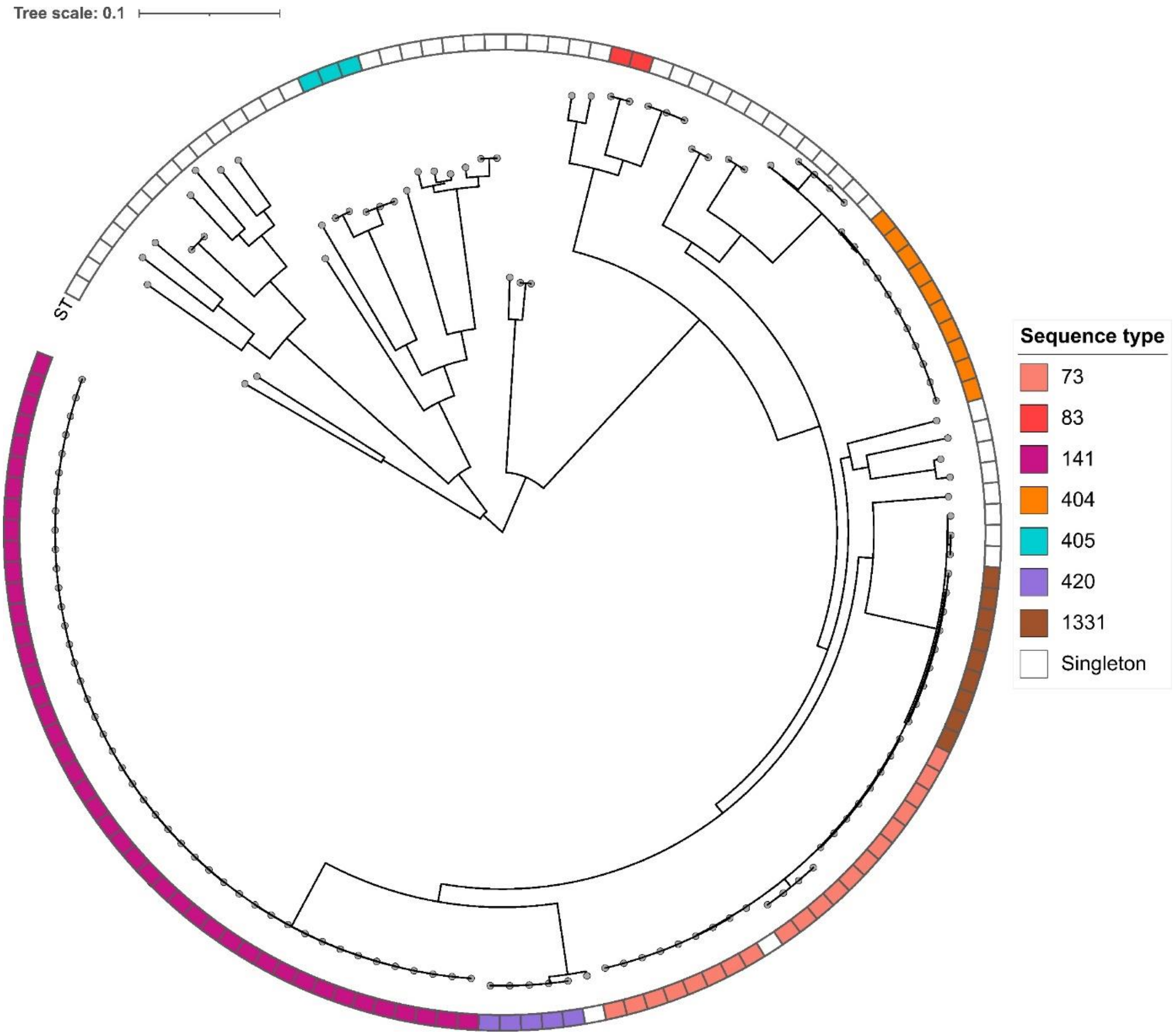

E.coli was the dominant MDRO+ species in our study, comprising several cluster contributing (colored) and non-cluster-contributing, i.e. “singletons” (white) isolates with respective sequence types (ST). ST141 was the dominant sequence type (n=28) that could further be differentiated into separate WGS-clusters.

© 2025 Nguyen T et al. *JAMA Network Open.*

eFigure 7. Cluster Attribution Comparison (AFLP vs WGS)

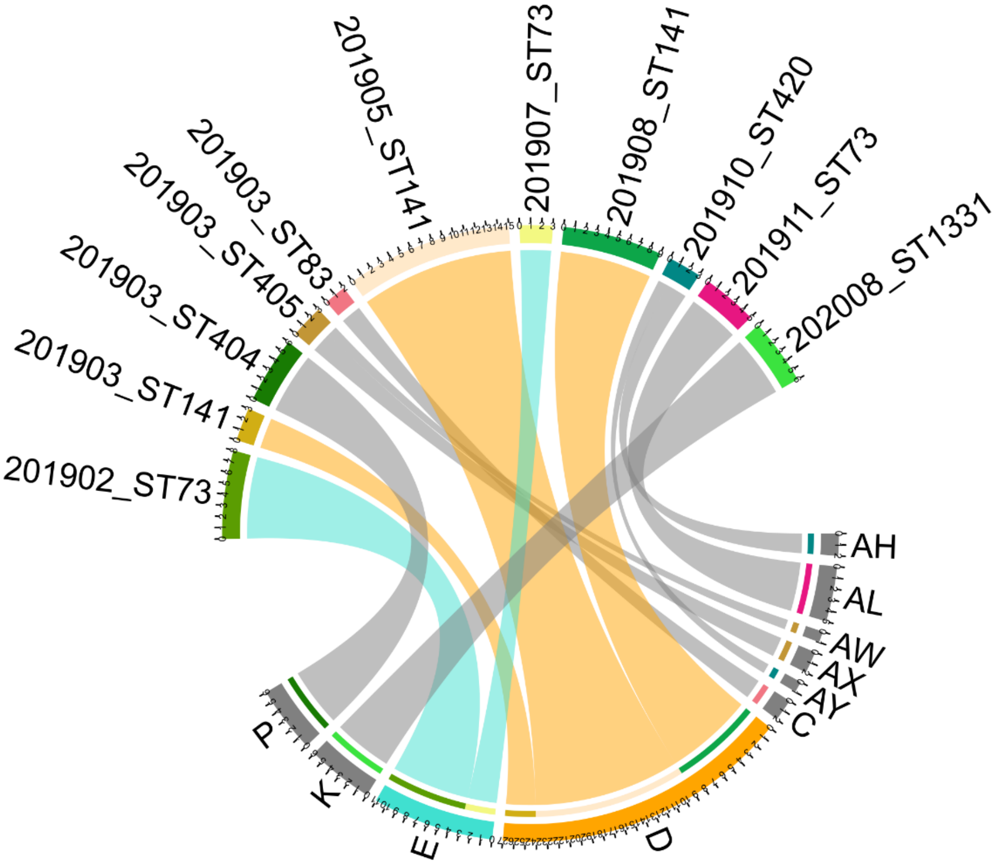

AFLP types (single- and dual-letters codes) were matched to their respective WGS clusters year/month/underscore/ST/Number) in the chord diagram. Allocation of one AFLP type to various WGS-clusters was defined as a mismatch. For example: Perceivable mismatches for AFLP type "D" (orange) and "E" (turquoise), where one AFLP type was attributed to multiple WGS-clusters; perceivable mismatches for AFLP-type "AY" and "AH", where both AFLP-types belonged to one WGS-cluster.

eFigure 8. Cluster Stratification by Sequence Type

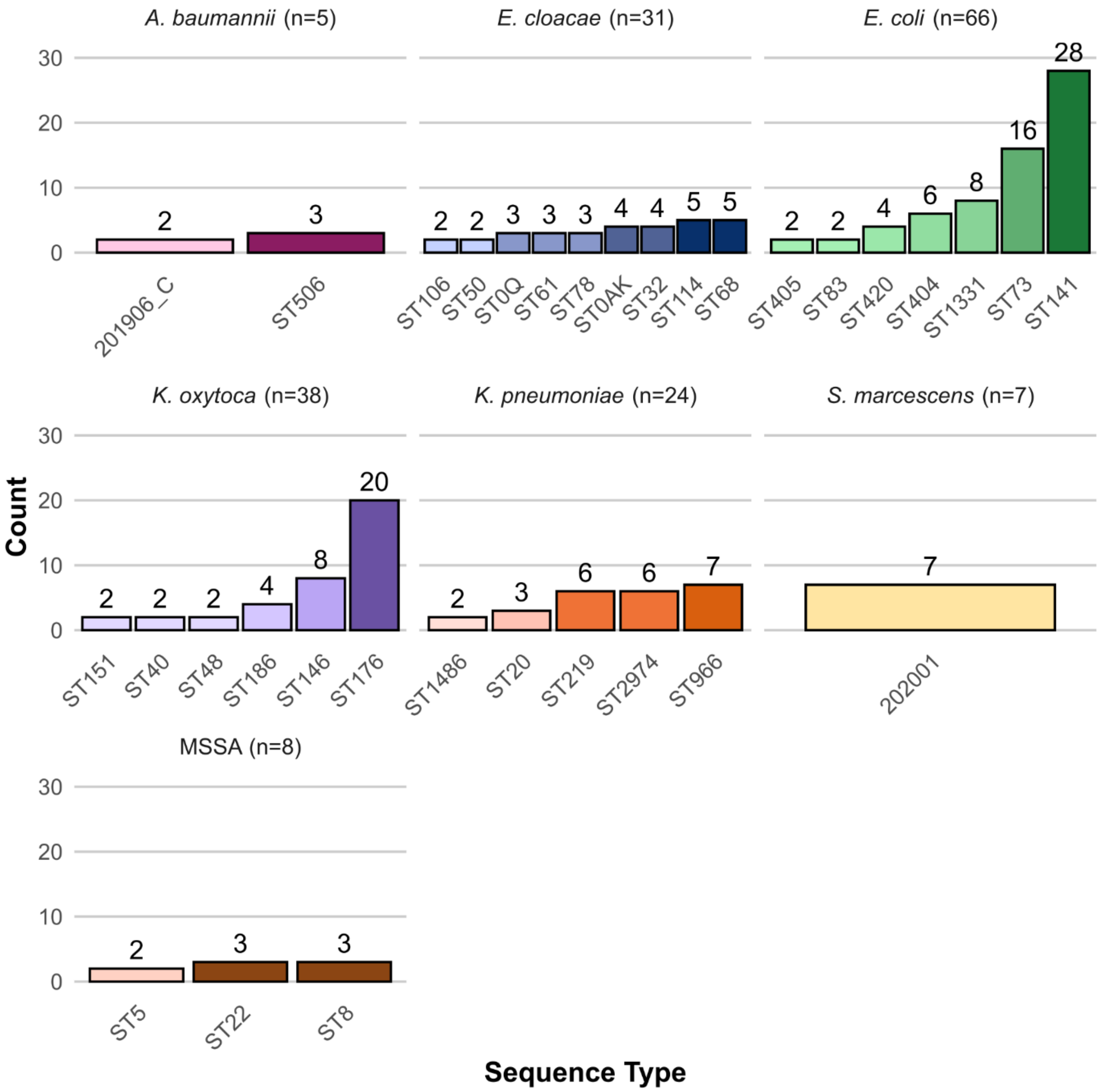

Stratification of cluster contributing MDRO+ isolates on sequence type level. The three most dominant species in our study were (descending): *E. coli*, *Klebsiella spp.* and *E. cloacae*.

© 2025 Nguyen T et al. *JAMA Network Open.*

eFigure 9. Alluvial Diagram of Observed MDRO+ Blood Stream Infections

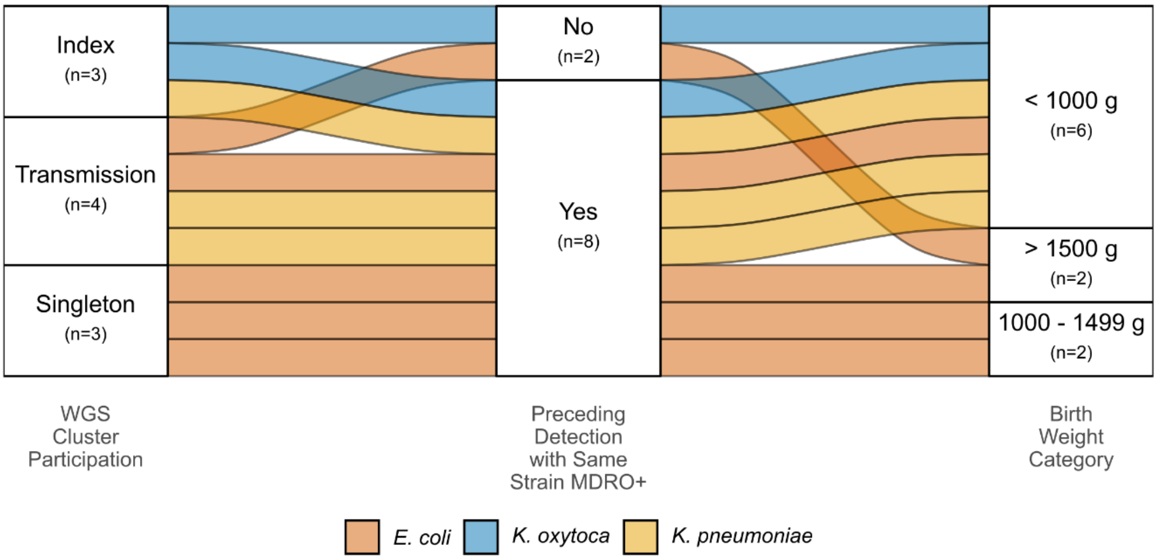

Every combination of three categories, indicated by connecting lines, resembles one patient. Seven BSI could be attributed to a TC as defined by WGS (Index, Transmission), of which 4 derived from a TE. Of these, three were preceded with the detection of same strain MDRO+ in materials other than blood culture samples.

© 2025 Nguyen T et al. *JAMA Network Open.*

eFigure 10. Antibiotic Administration by Birth Weight Groups

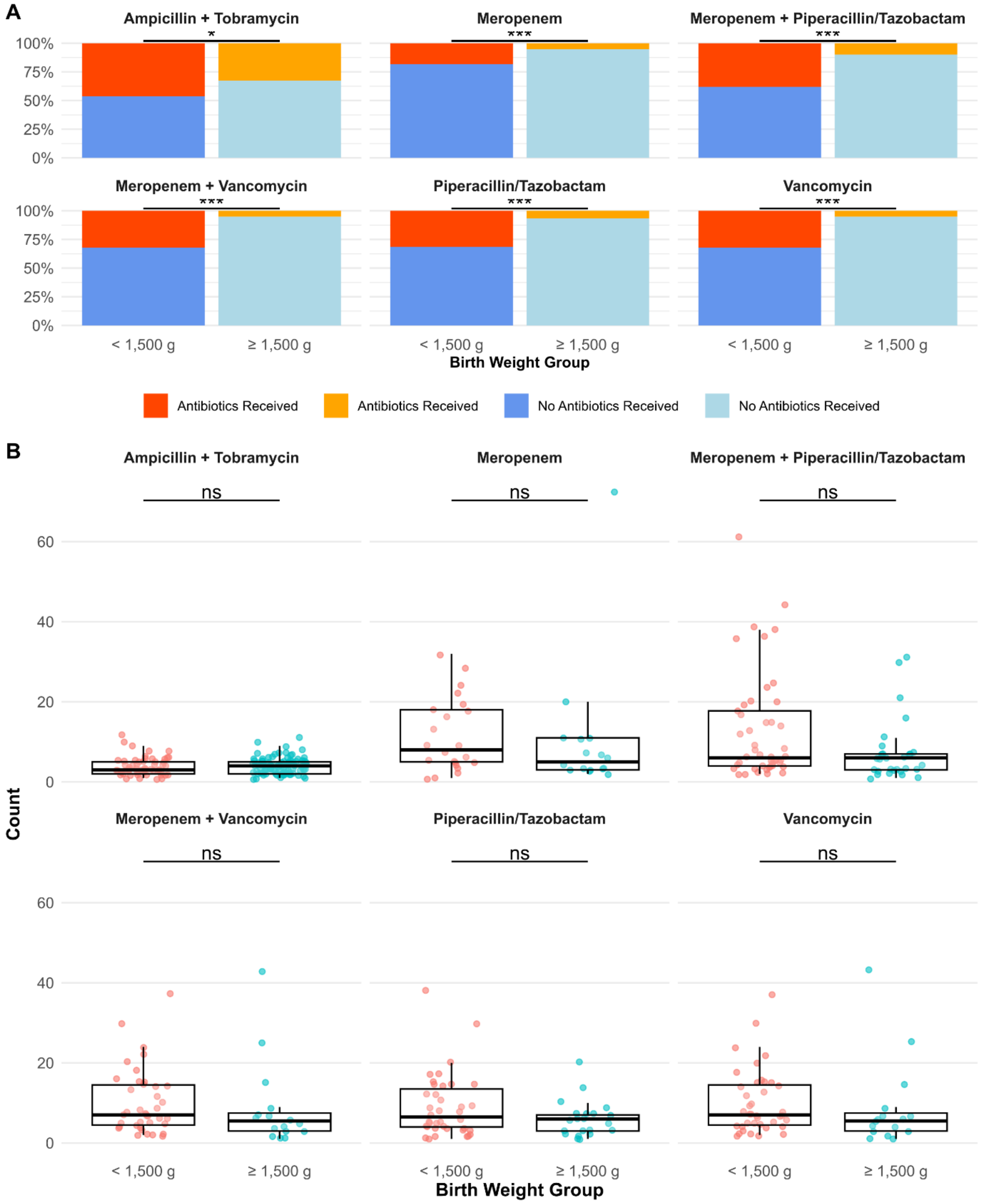

A) Proportion of patients treated with antibiotics or their combinations vs. patients that did not receive any antibiotics (stratified by birth weight groups). Proportionally, patients with a birth weight < 1,500 g receive the selected antibiotics or their combinations more often. Statistical significance according to Chi-Squared Test (* p < 0.05, ** p < 0.01, *** p < 0.001). **B)** Counts of antibiotics administered per patient, stratified by birth weight group. No statistical differences between groups and administered antibiotics or their combinations according to Wilcoxon Rank-Sum Test (ns = not significant).

© 2025 Nguyen T et al. *JAMA Network Open.*

eTable 1. Case-Report-Form

CRF	
ID	Vancomycin
PIZ (Pseudonym)	Tazobactam
Patient Number	Cotrim
Patient Name (Pseudonym)	Meropenem
Patient First Name (Pseudonym)	Cefuroxim
Date of Birth	Ampicillin
Sex	Tobramycin
Birth Weight	Erythromycin
Mode of Delivery	Amphotericin B
Multiple Birth	Rifampicin
Observation Date	Cefotaxim
Admission	Levofloxacin
Discharge	Teicoplanin
Type of Discharge	Linezolid
Room	Ceftazidim
Bed Location	Material
Incubator Number	Swab Site
Invasive Breathing	MDRO+
CPAP	Case Number (Microbiology)
CVC	Sample Number (Microbiology)
PVC	Date of Report (Microbiology)
UVC	Date of Sample Entry (Microbiology)
Arterial Line	Date of Report (AFLP)
Breast Milk	Internal Isolate Code
Feeding Tube	Resistence Category (KRINKO)
Bladder Catheter	Report Number (AFLP)
EVD	AFLP-Type
Kangarooing	Sequence Type (WGS)
INPULS Score	Cluster Definition (WGS)

Column headers of the Case-Report-Form.

© 2025 Nguyen T et al. *JAMA Network Open.*

eTable 2. Demographic Data of Study Population

	Overall		Combined: < 1,500 g		Combined: ≥ 1,500 g	
	N = 434		N = 121		N = 313	
Category	***Count (%)***	***95% CI***	***Count (%)***	***95% CI***	***Count (%)***	***95% CI***
Gender						
Female	192 (44.24)	39.52 - 49.06	52 (42.98)	34.11 - 52.29	140 (44.73)	39.16 - 50.43
Male	242 (55.76)	50.94 - 60.48	69 (57.02)	47.71 - 65.89	173 (55.27)	49.57 - 60.84
Birth Weight Category						
< 1000 g	64 (14.75)	11.62 - 18.52	64 (52.89)	43.64 - 61.96	0 (0)	NA
≥ 1000 g, < 1500 g	57 (13.13)	10.17 - 16.76	57 (47.11)	38.04 - 56.36	0 (0)	NA
≥ 1500 g, < 2500 g	146 (33.64)	29.25 - 38.33	0 (0)	0.00 - 3.8	146 (46.65)	41.04 - 52.34
≥ 2500 g	167 (38.48)	33.91 - 43.26	0 (0)	0.00 - 3.8	167 (53.35)	47.66 - 58.96
Delivery Mode						
NA	1 (0.23)	0.01 - 1.48	1 (0.83)	0.04 - 5.19	0 (0)	NA
C-section: Emergency	35 (8.06)	5.76 - 11.14	13 (10.74)	6.07 - 18.00	22 (7.03)	4.56 - 10.60
C-section: Primary	169 (38.94)	34.36 - 43.72	63 (52.07)	42.84 - 61.16	106 (33.87)	28.69 - 39.44
C-section: Secondary	91 (20.97)	17.29 - 25.17	30 (24.79)	17.60 - 33.62	61 (19.49)	15.34 - 24.41
Vaginal	138 (31.80)	27.48 - 36.44	14 (11.57)	6.70 - 18.97	124 (39.62)	34.20 - 45.29
Multiple Birth Mode						
Twins	79 (18.20)	14.75 - 22.23	27 (22.31)	15.46 - 30.97	52 (16.61)	12.76 - 21.31
Triplets	7 (1.61)	0.71 - 3.44	3 (2.48)	0.64 - 7.62	4 (1.28)	0.41 - 3.46
Quadriplets	3 (0.69)	0.18 - 2.18	3 (2.48)	0.64 - 7.62	0 (0)	NA
Single	345 (79.49)	75.32 - 83.13	88 (72.73)	63.75 - 80.24	257 (82.11)	77.31 - 86.10
Category	Median (Mean)	IQR (min - max)	Median (Mean)	IQR (min - max)	Median (Mean)	IQR (min - max)
Birth Weight (grams)	2,165 (2,215)	1,549 (405 - 4,870)	980 (1,019)	555 (405 - 1,490)	2,590 (2,678)	1,230 (1,520 - 4,870)
Gestational age (weeks)	34.6 (34.27)	6.9 (22.4 - 42.4)	28.7 (28.6)	4.6 (22.4 - 34.9)	36.7 (36.4)	5.14 (30.0 - 42.4)
Length of Stay (days)	8.0 (17.8)	15.0 (2.0 - 161.0)	29.0 (38.2)	43.0 (2.0 - 161.0)	6.0 (9.9)	7.0 (2.0 - 132.0)
MDRO+ Colonization Count per Patient (Percentage)						
0	209 / 434 (48%)		25 / 121 (21%)		184 / 313 (59%)	

© 2025 Nguyen T et al. *JAMA Network Open.*

1	112 / 434 (26%)	30 / 121 (25%)	82 / 313 (26%)
2	66 / 434 (15%)	35 / 121 (29%)	31 / 313 (9.9%)
3	28 / 434 (6.5%)	19 / 121 (16%)	9 / 313 (2.9%)
4	10 / 434 (2.3%)	8 / 121 (6.6%)	2 / 313 (0.6%)
5	4 / 434 (0.9%)	2 / 121 (1.7%)	2 / 313 (0.6%)
6	5 / 434 (1.2%)	2 / 121 (1.7%)	3 / 313 (1.0%)

The table divides the study population into a group with all enrolled patients ("Overall") and a group stratified by birth weight categories of < 1,500 g and ≥ 1,500 g.

© 2025 Nguyen T et al. *JAMA Network Open.*

eTable 3. WGS-Cluster-Contributing MDRO+

MDRO+	Sequence Type	WGS-Cluster	Number of Patients Involved	Max. Number of SNPs
A. baumannii	C	201906_C	2	3
A. baumannii	ST506	202006_fusA_SLV_ST506	3	1
E. cloacae	ST106	201903_ST106	2	0
E. cloacae	ST61	201904_ST61	3	7
E. cloacae	ST0Q	201907_ST0Q	3	2
E. cloacae	ST68	201907_ST68	5	7
E. cloacae	ST50	201911_ST50	2	1
E. cloacae	ST32	202001_ST32	4	4
E. cloacae	ST78	202002_ST78	3	1
E. cloacae	ST0AK	202005_ST0AK	4	1
E. cloacae	ST114	202006_ST114	5	2
E. coli	ST73	201902_ST73	8	4
E. coli	ST141	201903_ST141	3	0
E. coli	ST404	201903_ST404	6	3
E. coli	ST405	201903_ST405	2	1
E. coli	ST83	201903_ST83	2	3
E. coli	ST141	201905_ST141	15	3
E. coli	ST73	201907_ST73	3	3
E. coli	ST141	201908_ST141	10	2
E. coli	ST420	201910_ST420	4	4
E. coli	ST73	201911_ST73	5	2
E. coli	ST1331	202008_ST1331	8	2
K. oxytoca	ST176	201903_ST176	20	6
K. oxytoca	ST48	201909_ST48	2	0
K. oxytoca	ST40	201911_ST40	2	4
K. oxytoca	ST146	202002_ST146	8	4
K. oxytoca	ST151	202005_ST151	2	5
K. oxytoca	ST186	202005_ST186	4	7
K. pneumoniae	ST219	201908_ST219	6	5
K. pneumoniae	ST2974	201908_ST2974	6	2
K. pneumoniae	ST1486	201909_ST1486	2	5
K. pneumoniae	ST966	202006_ST966	7	6
K. pneumoniae	ST20	202008_ST20	3	0
MSSA	ST5	201907_ST5	2	9
MSSA	ST8	201910_ST8	3	3
MSSA	ST22	202002_ST22	3	1
S. marcescens	202001	202001	7	2
Total				179

MDRO+ species with respective ST, WGS-cluster-definition, numbers of patients involved (including index patients) and maximum number of SNPs between a cluster’s bacterial isolates.

© 2025 Nguyen T et al. *JAMA Network Open.*

eTable 4. WGS-non-Cluster-Contributing MDRO+ ("singletons")

MDRO+	Sequence Type	WGS-Cluster	Number of Patients Involved
A. baumannii	93	singleton	2
A. baumannii	106	singleton	1
A. baumannii	205	singleton	1
A. baumannii	ST1459	singleton	1
C. freundii	720	singleton	1
C. freundii	1262	singleton	1
C. freundii	162	singleton	1
C. freundii	64	singleton	1
C. freundii	98	singleton	1
E. cloacae	145	singleton	5
E. cloacae	dnaA_SLV_ST171	singleton	1
E. cloacae	novel profile 2	singleton	1
E. cloacae	218	singleton	1
E. cloacae	286	singleton	2
E. cloacae	1283	singleton	1
E. cloacae	158	singleton	1
E. cloacae	278	singleton	1
E. cloacae	664	singleton	1
E. cloacae	672	singleton	1
E. cloacae	78	singleton	1
E. cloacae	831	singleton	1
E. coli	69	singleton	5
E. coli	10	singleton	2
E. coli	127	singleton	2
E. coli	405	singleton	2
E. coli	428	singleton	2
E. coli	80	singleton	2
E. coli	95	singleton	2
E. coli	101	singleton	1
E. coli	1154	singleton	1
E. coli	117	singleton	1
E. coli	1193	singleton	1
E. coli	131	singleton	1
E. coli	1314	singleton	1
E. coli	14	singleton	1
E. coli	144	singleton	1
E. coli	2015	singleton	1
E. coli	2166	singleton	1
E. coli	23	singleton	1
E. coli	295	singleton	1
E. coli	38	singleton	1

© 2025 Nguyen T et al. *JAMA Network Open.*

E. coli	399	singleton	1
E. coli	3LV	singleton	1
E. coli	404	singleton	1
E. coli	569	singleton	1
E. coli	5910	singleton	1
E. coli	62	singleton	1
E. coli	657	singleton	1
E. coli	6949	singleton	1
E. coli	73	singleton	1
E. coli	967	singleton	1
E. coli	icd_SLV_ST73	singleton	1
K. aerogenes	857	singleton	1
K. aerogenes	253	singleton	1
K. aerogenes	93	singleton	1
K. aerogenes	135	singleton	1
K. oxytoca	675	singleton	1
K. oxytoca	676	singleton	1
K. oxytoca	234	singleton	1
K. oxytoca	40	singleton	2
K. oxytoca	11	singleton	1
K. oxytoca	13	singleton	1
K. oxytoca	176	singleton	1
K. oxytoca	178	singleton	1
K. oxytoca	184	singleton	1
K. oxytoca	36	singleton	1
K. oxytoca	64	singleton	1
K. oxytoca	84	singleton	1
K. oxytoca	88	singleton	1
K. oxytoca	673	singleton	1
K. oxytoca	rpoB_SLV_ST72	singleton	1
K. pneumoniae	200	singleton	2
K. pneumoniae	397	singleton	2
K. pneumoniae	45	singleton	2
K. pneumoniae	tonB_SLV_ST461	singleton	1
K. pneumoniae	8102	singleton	1
K. pneumoniae	105	singleton	1
K. pneumoniae	13	singleton	1
K. pneumoniae	1731	singleton	1
K. pneumoniae	1875	singleton	1
K. pneumoniae	1962	singleton	1
K. pneumoniae	2478	singleton	1
K. pneumoniae	26	singleton	1
K. pneumoniae	292	singleton	1

© 2025 Nguyen T et al. *JAMA Network Open.*

K. pneumoniae	308	singleton	1
K. pneumoniae	433	singleton	1
K. pneumoniae	465	singleton	1
K. pneumoniae	48	singleton	1
K. pneumoniae	596	singleton	1
K. pneumoniae	792	singleton	1
K. pneumoniae	883	singleton	1
P. aeruginosa	395	singleton	2
P. aeruginosa	108	singleton	1
S. marcescens	no_scheme	singleton	7
MSSA	398	singleton	5
MSSA	15	singleton	4
MSSA	30	singleton	3
MSSA	582	singleton	3
MSSA	7	singleton	3
MSSA	yqiL_SLV_ST7	singleton	1
MSSA	arcC(-)	singleton	1
MSSA	yqiL_SLV2_ST7	singleton	1
MSSA	1	singleton	2
MSSA	22	singleton	2
MSSA	45	singleton	2
MSSA	8	singleton	2
MSSA	946	singleton	2
MSSA	46	singleton	1
MSSA	5	singleton	1
MSSA	672	singleton	1
MSSA	72	singleton	1
MSSA	938	singleton	1
Total			157

MDRO+ species with respective ST, WGS-cluster-definition and numbers of patients involved.

© 2025 Nguyen T et al. *JAMA Network Open.*

eTable 5. AFLP-Mismatches for *E. coli*

E. coli	**AFLP-Type Matching WGS-Cluster**		**AFLP-Type does not match WGS-Cluster**	
**Clusters**	**AFLP Type**	**Count**	**AFLP Type**	**Count**
201902_ST73	***E***	8	-	-
201907_ST73	-	-	***E***	3
201911_ST73	AL	5	-	-
201903_ST404	P	6	-	-
201903_ST405	***AX***	2	***AW***	1
201903_ST83	C	2	-	-
201905_ST141	***D***	15	-	-
201908_ST141	-	-	***D***	9
201903_ST141	-	-	***D***	3
201910_ST420	***AH***	2	***AY***	1
202008_ST1331	K	6	-	-

Detailed allocation of AFLP-types to WGS-clusters for *E. coli*. Mismatched AFLP-Types in bold/cursive. AFLP-Types “D” and “E” successfully identified ST141 and ST73 respectively. WGS cluster definitions enabled further separation of *E. coli* Sequence Types. For example, ST 141 can be separated into distinct WGS clusters “201903_ST141” [3], “201905_ST141 [15], “201908_ST141 [10]”.

© 2025 Nguyen T et al. *JAMA Network Open.*

eTable 6. Cumulative AFLP-Mismatch Rates per MDRO+

**MDRO+**	**Cumulative Mismatch Rate (AFLP vs. WGS)**	**Number of WGS-Clusters**
A. baumannii	0,00	2
E. cloacae	0,09	9
E. coli	0,06	11
K. oxytoca	0,10	6
K. pneumoniae	0,08	5
S. marcescens	0,00	1
MSSA	0,11	3

Based on the less granular resolution of AFLP-typing in comparison to WGS we observed cumulative mismatch rates for respective MDRO+ species. Note: S. *aureus* underwent *spa*-typing as AFLP-typing is inadequate for this species.

© 2025 Nguyen T et al. *JAMA Network Open.*

eTable 7. WGS Enabled Net Gain in Resolution

Cluster (Species Level)	Cluster Start	Cluster End	WGS Clusters with Overlapping Timeframe	Resolution Gain
A. baumannii 1	2019-06-10	2019-07-29	201906_C	0
A. baumannii 7	2020-06-01	2020-08-02	202006_fusA_SLV_ST506	0
E. cloacae 2	2019-03-03	2019-03-10	201903_ST106	0
E. cloacae 3	2019-04-15	2019-04-15	201904_ST61	0
E. cloacae 4	2019-04-22	2019-04-22	201904_ST61	0
E. cloacae 5	2019-06-16	2019-09-01	201907_ST0Q, 201907_ST68	1
E. cloacae 6	2019-09-22	2019-12-16	201911_ST50	0
E. cloacae 7	2020-01-22	2020-03-01	201911_ST50, 202001_ST32, 202002_ST78	2
E. cloacae 8	2020-03-15	2020-04-26	202001_ST32, 202002_ST78	1
E. cloacae 9	2020-05-10	2020-05-10	202005_ST0AK	0
E. cloacae 10	2020-05-17	2020-06-28	202005_ST0AK, 202006_ST114	1
E. coli 2	2019-03-03	2020-03-08	201902_ST73, 201903_ST141, 201903_ST404, 201903_ST405, 201903_ST83, 201905_ST141, 201907_ST73, 201908_ST141, 201910_ST420, 201911_ST73	9
E. coli 12	2020-08-10	2020-10-25	202008_ST1331	0
K. oxytoca 5	2019-09-05	2019-09-16	201909_ST48	0
K. oxytoca 11	2020-02-16	2020-04-26	202002_ST146	0
K. oxytoca 12	2020-05-10	2020-10-25	201903_ST176, 201911_ST40, 202005_ST151, 202005_ST186	3
K. oxytoca 13	2020-11-01	2020-11-15	201903_ST176	0
K. pneumoniae 7	2019-08-25	2020-02-05	201908_ST219, 201908_ST2974, 201909_ST1486	2
K. pneumoniae 11	2020-05-10	2020-10-26	202006_ST966, 202008_ST20	1
MSSA 1	2019-02-17	2019-09-08	201907_ST5	0
MSSA 3	2019-10-14	2020-03-08	201910_ST8, 202002_ST22	1
MSSA 4	2020-03-15	2020-10-18	202002_ST22	0
S. marcescens 6	2020-01-06	2020-01-06	202001	0
S. marcescens 7	2020-01-12	2020-03-01	202001	0

Table of species-level clusters that overlap with WGS clusters in time and the gain in cluster-resolution. The day of detection of an MDRO+ defines the start date. The discharge of the last patient to be colonized with respective cluster MDRO+ defines the end date. This logic applies for both clusters on species and WGS level.

© 2025 Nguyen T et al. *JAMA Network Open.*

eAppendix. Abbreviations

ESBL	Extended spectrum betalactamase
MRSA	Methicillin resistant *Staphylococcus aureus*
MALDI-TOF	Matrix-assisted-laser desorption-ionization time-of-flight
MIC	Minimum inhibitory concentration
EUCAST	European Committee on Antimicrobial Susceptibility Testing
MDRO+	Multidrug resistant organism plus
MSSA	Methicillin susceptible *Staphylococcus aureus*
G-BA	Gemeinsamer Bundesausschuss
AFLP	Amplified fragment length polymorphism
WGS	Whole genome sequencing
DNA	Deoxyribonucleic acid
PCR	Polymerase chain reaction
MLST	Multilocus sequence typing
SNP	Single-nucleotide polymorphism
TE	Transmission Event
MA	Moving averages
AIC	Akaike's information criterion
VIF	Variance inflation factor
NA	Not available

© 2025 Nguyen T et al. *JAMA Network Open.*

eReferences.

[1] Götting T, Reuter S, Jonas D, Hentschel R, Henneke P, Klotz D, et al. Protocol for a prospective cohort study: Prevention of Transmissions by Effective Colonisation Tracking in Neonates (PROTECT-Neo). BMJ Open 2020;10:e034068. https://doi.org/10.1136/bmjopen-2019-034068.

[2] Gemeinsamer Bundesausschuss. Beschluss des Gemeinsamen Bundesausschusses über eine Änderung der Qualitätssicherungsrichtlinie Früh- und Reifgeborene/QFR-RL: Erstfassung der Datenfelder der Strukturabfrage als Anlage 6. Bundesanzeiger. 2019 May 28. Available from: https://www.g-ba.de/downloads/39-261-3581/2018-11-22_QFR-RL_Erstfassung-Anlage-6_BAnz.pdf. n.d.

[3] Jonas D, Spitzmüller B, Daschner FD, Verhoef J, Brisse S. Discrimination of Klebsiella pneumoniae and Klebsiella oxytoca phylogenetic groups and other Klebsiella species by use of amplified fragment length polymorphism. Research in Microbiology 2004;155:17–23. https://doi.org/10.1016/j.resmic.2003.09.011.

[4] Jonas D, Speck M, Daschner FD, Grundmann H. Rapid PCR-Based Identification of Methicillin-Resistant *Staphylococcus aureus* from Screening Swabs. J Clin Microbiol 2002;40:1821–3. https://doi.org/10.1128/JCM.40.5.1821-1823.2002.

[5] Ponstingl H, Ning Z. SMALT - A new mapper for DNA sequencing reads. Wellcome Trust Sanger Institute; 2010. Available from: ftp://ftp.sanger.ac.uk/pub/hp3/smalt.tgz. DOI: not available.

[6] Danecek P, Bonfield JK, Liddle J, Marshall J, Ohan V, Pollard MO, et al. Twelve years of SAMtools and BCFtools. GigaScience 2021;10:giab008. https://doi.org/10.1093/gigascience/giab008.

[7] McKenna A, Hanna M, Banks E, Sivachenko A, Cibulskis K, Kernytsky A, et al. The Genome Analysis Toolkit: A MapReduce framework for analyzing next-generation DNA sequencing data. Genome Res 2010;20:1297–303. https://doi.org/10.1101/gr.107524.110.

[8] Bankevich A, Nurk S, Antipov D, Gurevich AA, Dvorkin M, Kulikov AS, et al. SPAdes: A New Genome Assembly Algorithm and Its Applications to Single-Cell Sequencing. Journal of Computational Biology 2012;19:455–77. https://doi.org/10.1089/cmb.2012.0021.

[9] Wood DE, Salzberg SL. Kraken: ultrafast metagenomic sequence classification using exact alignments. Genome Biol 2014;15:R46. https://doi.org/10.1186/gb-2014-15-3-r46.

[10] Seemann T. MLST: Multi-locus sequence typing. [software on the internet]. GitHub; 2017 [cited 2024 Nov 18]. Available from: https://github.com/tseemann/mlst.

[11] David S, Reuter S, Harris SR, Glasner C, Feltwell T, Argimon S, et al. Epidemic of carbapenem-resistant Klebsiella pneumoniae in Europe is driven by nosocomial spread. Nat Microbiol 2019;4:1919–29. https://doi.org/10.1038/s41564-019-0492-8.

[12] Coll F, Harrison EM, Toleman MS, Reuter S, Raven KE, Blane B, et al. Longitudinal genomic surveillance of MRSA in the UK reveals transmission patterns in hospitals and the community. Sci Transl Med 2017;9:eaak9745. https://doi.org/10.1126/scitranslmed.aak9745.

[13] Barnett A, Graves N. Competing risks models and time-dependent covariates. Crit Care 2008;12:134. https://doi.org/10.1186/cc6840.

[14] Breslow N, Day N. Statistical methods in cancer research. 2: The design and analysis of cohort studies. Reprinted. Lyon: 1991.

[15] Utts JM. The rainbow test for lack of fit in regression. Communications in Statistics - Theory and Methods 1982;11:2801–15. https://doi.org/10.1080/03610928208828423.

[16] Krämer W, Sonnberger H. The Linear Regression Model Under Test. Heidelberg: Physica-Verlag HD; 1986. https://doi.org/10.1007/978-3-642-95876-2.

[17] Box GEP, Cox DR. An Analysis of Transformations. Journal of the Royal Statistical Society: Series B (Methodological) 1964;26:211–43. https://doi.org/10.1111/j.2517-6161.1964.tb00553.x.

[18] Yeo I-K. A new family of power transformations to improve normality or symmetry. Biometrika 2000;87:954–9. https://doi.org/10.1093/biomet/87.4.954.

© 2025 Nguyen T et al. *JAMA Network Open.*
