# Supplementary material for: Prospective Whole-Genome Sequencing to Identify Bacterial Transmission and Its Modifiers in Neonates
Source: JAMA Netw Open. 2025 Nov 21;8(11):e2541409. doi: 10.1001/jamanetworkopen.2025.41409 (PMC12639485; doi:10.1001/jamanetworkopen.2025.41409)
Supplement: Supplement 3. — Data Sharing Statement [file jamanetwopen-e2541409-s003.pdf]

Data Sharing Statement

Nguyen. Prospective Whole-Genome Sequencing to Identify Bacterial Transmission and Its Modifiers in Neonates. *JAMA Netw Open*. Published November 21, 2025.
doi:10.1001/jamanetworkopen.2025.41409

Data
Data available: Yes
Data types: Deidentified participant data
How to access data: philipp.henneke@uniklinik-freiburg.de
When available: With publication

Supporting Documents
Document types: None

Additional Information
Who can access the data: Researchers whose proposed use of the data has been approved
Types of analyses: Comparison with own cohorts
Mechanisms of data availability: With signed data access agreement
